# Structural basis for fragmenting the exopolysaccharide of *Acinetobacter baumannii* by bacteriophage ΦAB6 tailspike protein

**DOI:** 10.1038/srep42711

**Published:** 2017-02-17

**Authors:** I-Ming Lee, I-Fan Tu, Feng-Ling Yang, Tzu-Ping Ko, Jiahn-Haur Liao, Nien-Tsung Lin, Chung-Yi Wu, Chien-Tai Ren, Andrew H.-J. Wang, Ching-Ming Chang, Kai-Fa Huang, Shih-Hsiung Wu

**Affiliations:** 1 Institute of Biochemical Sciences, National Taiwan University, Taipei 106, Taiwan; 2 Institute of Biological Chemistry, Academia Sinica, Taipei 115, Taiwan; 3 Core Facilities for Protein Structural Analysis (CFPSA), Academia Sinica, Taipei 115, Taiwan; 4 Master program in Microbiology and Immunology, Tzu Chi University, Hualien 970, Taiwan; 5 Genomics Research Center, Academia Sinica, Taipei 115, Taiwan; 6 Department of Chemistry, National Taiwan University, Taipei 106, Taiwan

## Abstract

With an increase in antibiotic-resistant strains, the nosocomial pathogen *Acinetobacter baumannii* has become a serious threat to global health. Glycoconjugate vaccines containing fragments of bacterial exopolysaccharide (EPS) are an emerging therapeutic to combat bacterial infection. Herein, we characterize the bacteriophage ΦAB6 tailspike protein (TSP), which specifically hydrolyzed the EPS of *A. baumannii* strain 54149 (*Ab-54149). Ab-54149* EPS exhibited the same chemical structure as two antibiotic-resistant *A. baumannii* strains. The ΦAB6 TSP-digested products comprised oligosaccharides of two repeat units, typically with stoichiometric pseudaminic acid (Pse). The 1.48-1.89-Å resolution crystal structures of an N-terminally-truncated ΦAB6 TSP and its complexes with the semi-hydrolyzed products revealed a trimeric β-helix architecture that bears intersubunit carbohydrate-binding grooves, with some features unusual to the TSP family. The structures suggest that Pse in the substrate is an important recognition site for ΦAB6 TSP. A region in the carbohydrate-binding groove is identified as the determinant of product specificity. The structures also elucidated a retaining mechanism, for which the catalytic residues were verified by site-directed mutagenesis. Our findings provide a structural basis for engineering the enzyme to produce desired oligosaccharides, which is useful for the development of glycoconjugate vaccines against *A. baumannii* infection.

The gram-negative pathogen *Acinetobacter baumannii (Ab*) causes nosocomial urinary tract and bloodstream infections, ventilator-associated pneumonia, and meningitis^1^. In the past decade, estimated mortality of hospital outbreaks and community-acquired *Ab* infections was 26% to 68%^2^. While carbapenem and colistin are used in first-line treatments^3^, antibiotic-resistant strains are emerging^2^, likely due to this pathogen acquiring antibiotic-resistant genes from other *Acinetobacter* strains or from bacteria colonizing the same environment through horizontal gene transfer^4^. This represents a rigorous challenge to the treatment of *Ab* infections in the future, so there is a pressing need for the development of new therapies.

In addition to antibiotic treatments, another choice for combating bacterial infections is vaccination, particularly utilizing the bacterial exopolysaccharide (EPS) to generate glycovaccines^5^,^6^. Glycoconjugate vaccines (GCV), which are generated by conjugating the bacterial EPS to a carrier protein, can elicit a strong and long-lasting immune response compared to vaccines using whole polysaccharide^7^,^8^. This is likely due to GCVs preferentially stimulating the adaptive immune responses^9^,^10^,^11^. However, the heterogeneity of bacterial EPS extracts hampered the development of GCVs. The heterogeneous mixture of polymerization forms of EPS usually resulted in multipoint attachment of protein on the polysaccharide when conjugating to carrier proteins, leading to a considerable challenge to the reproducibility of glycoconjugates. It has been well known that many bacteriophages are capable of depolymerizing the EPS of their host bacteria through the tailspike proteins (TSPs)^12^. The digested products comprise up to two oligosaccharides with some repeat units in general^13^. In this way, TSPs can be employed to make EPS derivatives with preferred size and homogeneity (Supplementary Fig. 1).

Bacteriophage TSP is found at the tail of the viral particle^12^. They comprise elongated monomers with β-helical topology, which associate into trimers. These proteins bind to the ESP of specific bacteria and digest the EPS to facilitate the attachment of virions to the host cells. Previous studies of TSP structures, such as TSPs from bacteriophages P22, Sf6 and HK620, including oligosaccharide complexes have shed light on the substrate binding modes and catalytic mechanisms^14^,^15^,^16^. Here we present the crystal structures of the TSP from bacteriophage ΦAB6 and its complex with cleaved EPS fragments of *Ab-*54149, a clinical *A. baumannii* strain in Taiwan. ΦAB6 was reported to belong to the *Autographivirinae* subfamily and to specifically recognize *Ab-*54149^17^. In the present study, the substrate binding mode and catalytic mechanism of ΦAB6 TSP were subsequently elucidated by structural analysis and site-specific mutagenesis. These results can serve as a basis for producing better EPS fragments as the starting materials of GCV.

## Results

### Characterization of ΦAB6 TSP

**Figure 1:**
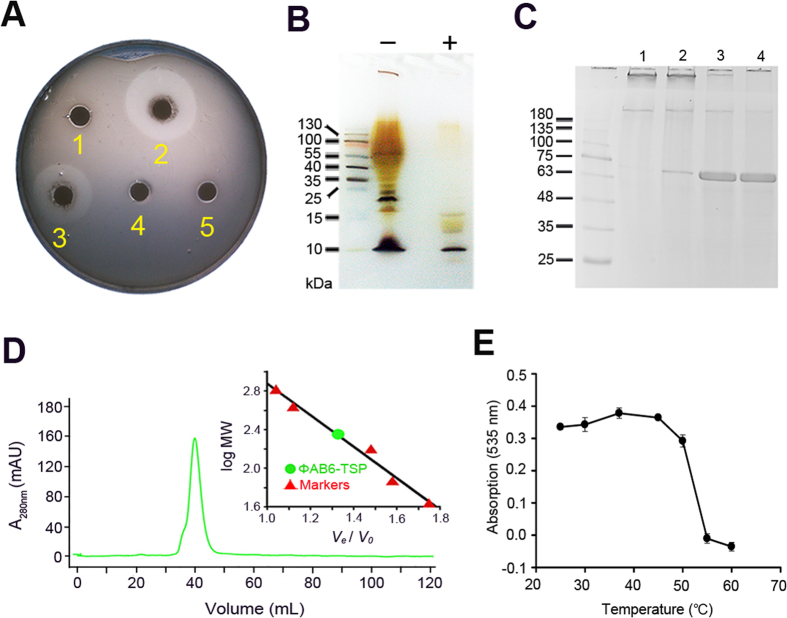
Characterization of the recombinant ΦAB6 TSP. (**A**) Evaluation of enzyme activity of wild-type and mutant ΦAB6 TSPs using top agar assay. The top agar was pre-inoculated with *Ab*-54149 and then spotted with (1) buffer (25 mM Tris-HCl, 0.1 M NaCl, pH 7.5), (2) wild-type ΦAB6 TSP, (3) ΦAB6 TSPΔN, (4) mutant E425Q, and (5) mutant E447Q. (**B**) Analysis of the extract of *Ab*-54149 exopolysaccharide by silver-stained SDS-PAGE. About 150 μg exopolysaccharide extract with (+) or without (−) the treatment of ΦAB6 TSP was loaded into the gel. The first lane was loaded with molecular weight markers. (**C**) Ability of resistance to the SDS denaturation. The enzyme was pre-heated at 80 °C for 0 (lane 1), 1 (lane 2), 5 (lane 3), and 10 (lane 4) min in the presence of 2% SDS. (**D**) Oligomerization status in solution. ΦAB6 TSP was eluted as a single peak from an analytical gel-filtration column. Based on the calibration curve of log MW versus V_e_/V_0_ (V_e_, elution volume; V_0_, void volume) generated with five protein markers (the inset), the elution volume of ΦAB6 TSP corresponds to a molecular weight of 217.9 kDa, very close to the theoretic value (228.5 kDa) of the trimer calculated from the amino acid sequence. (**E**) Evaluation of thermal stability. The enzyme was heated at varied temperatures for 5 min and then the enzyme activity was evaluated immediately as described in the Methods. The experiment was performed in duplicates.

The open reading frame encoding the tailspike protein of ΦAB6 (ΦAB6 TSP) has recently been identified^17^. Here, a recombinant ΦAB6 TSP was overexpressed in *E. coli* cells and purified to near homogeneity. The purified ΦAB6 TSP was shown to generate a translucent halo when spotted on a top agar inoculated with *A. baumannii* strain 54149 (*Ab*-54149). The enzyme also degraded the extracts of *Ab*-54149 exopolysaccharide (EPS) in a time-dependent manner (Fig. 1A and Supplementary Fig. 2A), manifesting its catalytic activity. As analyzed by silver-stained SDS-PAGE, ΦAB6 TSP appeared to specifically degrade the lipopolysaccharide of *Ab*-54149 (Fig. 1B), presumably to the O-antigen^18^. Similar to the reported bacteriophage TSPs^13^,^14^,^15^,^16^, ΦAB6 TSP was shown to be homotrimeric in solution and resistant to SDS denaturation (Fig. 1C and D and Supplementary Fig. 2B). By contrast, its thermal stability (*T*_m_ ~55 °C) was relatively low compared to other TSPs (Fig. 1E and Supplementary Fig. 2C)^14^.

### Characterization of hydrolyzed products

**Figure 2:**
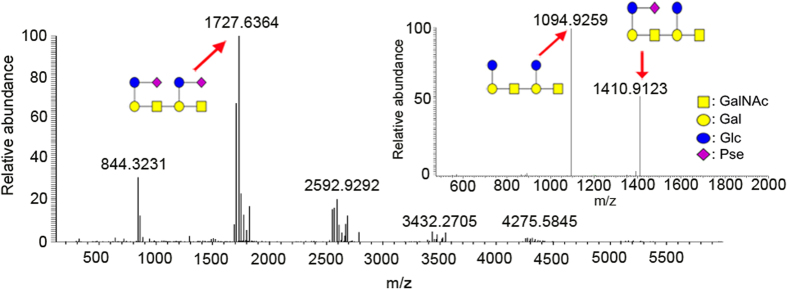
ESI-MS analysis of the ΦAB6 TSP-digested products. The major peak in the ESI-MS spectrum, as indicated by a red arrow, which corresponds to an m/z value of 1727.6, was subjected to ESI-MS-MS analysis (the inset). The results indicated that the main digestion product comprises oligosaccharide of two repeat units with two pseudaminic acids, as described in the text.

We analyzed the whole extract and ΦAB6 TSP-digested products of *Ab*-54149 EPS by 1D (^1^H and ^13^C) and 2D NMR (COSY, HSQC and HMBC). The results indicated the EPS to be made up of repeat units of → 3)-β-GalNAc*p*-(1 → 3)-[β-Glc*p*-(1 → 6)]-β-Gal*p*-(1 → , and a majority of the repeat units contained a pseudaminic acid (Pse) connected to Glc*p* via a β-Pse-(2 → 6)-β-Glc*p* linkage (Supplementary Figs 3~8, Supplementary Table 1). The hydrolyzed products were further analyzed by LC-ESI-MS, which revealed a major product (m/z = 1727.6324) of two repeat units with two Pse residues (Fig. 2). Moreover, the MS spectra also showed two minor peaks (m/z = 1411.50 and 1095.51) corresponding to the fragments of two repeat units, one with a Pse and other without Pse, (Supplementary Fig. 9), in agreement with the findings from our NMR assignment.

### Overall structure of ΦAB6 TSP∆N

**Figure 3:**
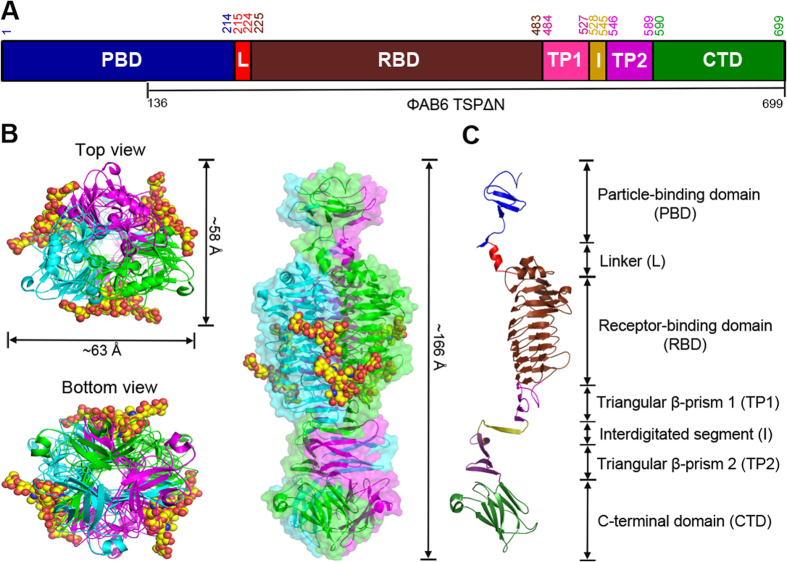
Overall structure. (**A**) Domains in full-length ΦAB6 TSP are shown in a schematic representation. The numberings for amino acid residues at the start and end of each domain are further indicated. TSP∆N includes residues 136–699. (**B**) The TSP∆N trimer is shown as three-colored ribbons in three different views, with bound oligosaccharides shown as space-filled models. The view on the right is perpendicular to the trimer axis onto the intersubunit groove with the N-terminal end on top, and a transparent surface of the structure is shown as well. **(C)** The seven domains in a TSP∆N monomer are colored as in **(A)**.

Despite testing numerous crystallization conditions, we did not obtain suitable crystals using the full-length ΦAB6 TSP. This is most likely due to the flexible linker between its N-terminal particle-binding domain and the central receptor-binding domain, as described for other TSPs^14^,^15^,^16^.We therefore produced an N-terminally-truncated form (residues 136–699, named ΦAB6 TSP∆N) based on sequence alignment with other TSPs (Fig. 3A). ΦAB6 TSP∆N retained nearly all of the enzymatic activity, homotrimeric assembly, and thermal stability of ΦAB6 TSP (Fig. 1A and Supplementary Fig. 2B and C). ΦAB6 TSP∆N was then crystallized successfully and the crystal structure was solved by the SAD-phasing method via Se-labeled ΦAB6 TSP∆N and refined to 1.48-Å resolution, with one ΦAB6 TSP∆N trimer in the asymmetric unit (Table 1). The trimeric ΦAB6 TSP∆N is a compact, elongated molecule with an overall length of ~166 Å and the diameters between 26 and 63 Å (Fig. 3B). The three subunits are tightly packed together to form a parallel, left-handed, superhelical twist. Each subunit exhibits a parallel β-helix in the main body and can be divided into the N-terminal particle-binding domain (PDB), the linker region (L), the receptor-binding domain (RBD), two triangular β-prisms (TP1 and TP2) connected by a highly interdigitated segment (I), and the C-terminal domain (CTD) (Fig. 3C).

### Structure of the ΦAB6 TSP∆N monomer

The N-terminal PBD region (Ser136-Ile214) of each subunit comprises a three-stranded, antiparallel β-sheet flanked by twisted loops and a β-turn. It is followed by a β-hairpin of ~16 Å in length. The loop connecting the second and third strands extrudes prominently from the β-sheet and contacts the N-terminal loops via two hydrogen bonds (H-bonds) (Fig. 3C). The N-terminal region appears to be the most flexible part as judged by the high average B factor (19.7 Å^2^) compared to that of the whole subunit (14.6 Å^2^). This likely led to the broken electron densities around the N-terminal 18 residues in all subunits. The β-hairpin is succeeded by a short α-helix (Thr215-Ile224) linking PBD and RBD. This linker is a common motif in the TSP structures reported thus far^14^,^15^,^16^.

**Figure 4:**
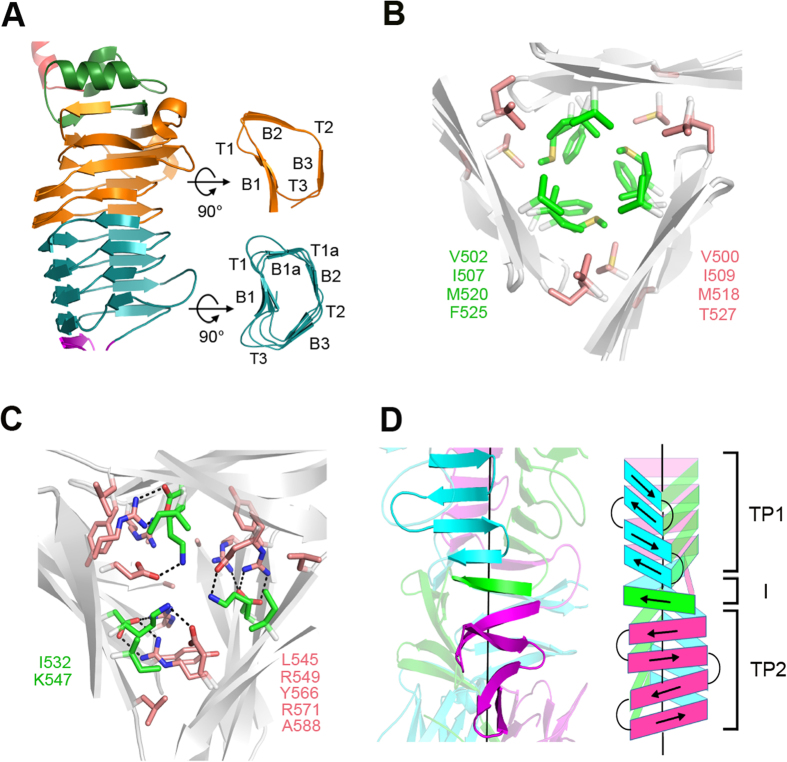
Structures of the β-helix domain and the triangular β-prisms. (**A)** The central, right-handed, parallel β-helix in ΦAB6 TSP∆N monomer. The first four rungs, the last four rungs, and the extended α,β-mixed turn on top are colored orange, deep teal, and green, respectively. (**B**) The interior of triangular β-prism 1 (TP1). The aliphatic side chain stacks at the center and the corners are colored green and salmon, respectively. The residues participating in the stacks are indicated on both sides. (**C**) The interior of the interdigitated segment and TP2. The black dotted lines depict the side chain hydrogen bonds between these residues. (**D**) TP1 and TP2 are connected by a highly interdigitated segment (I). The interdigitated segment merges into the β-sheets of TP1 and TP2 from different neighboring subunits, resulting in the formation of a nine-stranded, highly twisted, mixed β-sheet, as illustrated by cartoon on the right. The three subunits in ΦAB6 TSP∆N are colored cyan, green, and magenta.

The central RBD region (Thr225-Ser483) folds into a right-handed, parallel β-helix composed of 8 complete rungs, with an extended α,β-mixed turn on top, where a 10-residue α-helix caps the hydrophobic core of β-helix domain. This “cap” is preceded by a 3_10_-helix and an α-turn (Fig. 3C). The main body of the β-helix is collapsed to an L-shaped (rungs 1–4) or a kidney-shaped (rungs 5–8) cross section with the first complete rung starting at Val270. Rungs 1–4 are organized into three strands, B1, B2, and B3, separated by turns T1, T2, and T3, as seen in other TSPs, while rungs 5–8 contain an extra strand (B1a) within the T1 region (Fig. 4A). The extra strands were also identified in the β-helix structures of several galacturonases^19^, but not in other TSPs (Supplementary. Fig. 10). The three sets of parallel B1, B2, and B3 strands merge into the b1, b2 and b3 β-sheets, respectively, and form the three faces of the β-helix domain. In the 8 complete rungs, turns T1 and T3 vary in length ranging between 2 and 10 residues and, together with strands B1, form a concave solvent-exposed groove (Fig. 4A). T3 of rung 1 contains a 5-residue 3_10_-helix. By contrast, the length of T2 is restricted to 2 residues, except for the second T2, which contains 14 residues, protrudes from the border of the β-helix, and forms a 7-residue α-helix near the B3. The hydrophobic interior of the β-helix contains only two water molecules bound to the side chains of Gln355 and Ser375 and the main chains of Leu372, Ser375, Gly405, and His406. Moreover, there are a number of inward-orientated side chain stacks of cysteine (Cys417/Cys439), isoleucine (e.g. Ile335/Ile356/Ile380), valine (Val414/Val436), leucine (e.g. Leu349/Leu372), phenylalanine (e.g. Phe395/Phe419/Phe441/Phe471), and mixed-type amino acids (e.g.Phe243/Val270/Phe310/Ile335/Ile356/Ile380/Met408/Ile431/ Leu458). These aliphatic and aromatic stacks, together with 116 H-bonds, presumably stabilize the β-helical conformation.

Lys484-Pro589 is a β-sheet region that forms the triangular β-prisms in the trimer. The polypeptide chain makes a nearly U-shaped turn towards the C-terminus and then folds into a four-stranded antiparallel β-sheet. This β-sheet extends β-sheet b of the β-helix domain towards the C-terminus. After this β-sheet, the polypeptide chain loops out with respect to the subunit longitudinal axis, makes a ~90° bend and a sharp turn, and then forms the second four-stranded antiparallel β-sheet ~22 Å away from the first four-stranded β-sheet, with a nearly parallel orientation. The segment (Gly528-Leu545) connecting the two four-stranded β-sheets tightly interdigitates with the neighboring subunits once the monomers assemble into a trimer as described below. In the CTD region (Met590 -Ser699), the polypeptide chain is rotated by ~60° around the longitudinal axis with respect to the second four-stranded β-sheet, tilted toward the axis by ~30°, and organized as an independent β-sandwich formed by three-stranded and four-stranded antiparallel β-sheets. A short α-helix is included in the connecting loop between the fifth and sixth strands.

### Structure of the ΦAB6 TSP∆N trimer

In a trimer, three closely-packed subunits are related by a non-crystallographic triad, and ~39.8% (11453 out of 28710 Å^2^) of the solvent-accessible surface area in each subunit is buried. In the N-terminal PBD region, three β-sheets, each from a different subunit, are laterally associated and stabilized by side chain H-bonds. The N-terminal twisted loops project into the other subunits and further strengthen the subunit association. Moreover, the β-hairpin of each subunit inserts into a cleft of its neighboring subunit, formed by the “linker” and “cap” α-helices and a 3_10_-helix on top of β-helix domain, and makes extensive contact between the subunits. In the linker region, three α-helices from three different subunits form a helix bundle stabilized by 6 sidechain H-bonds between the three pairs of Gln216 and Asn220, rather than hydrophobic contacts observed in other TSP structures^14^,^15^,^16^. In RBD, the three β-helices are packed laterally through the b2 and b3 β-sheets to form a parallel, left-handed superhelix. At the interface of two adjacent β-helices, 35 residues are involved in the intersubunit interactions, of which 58% are H-bonds, 11% salt bridges, and 21% hydrophobic interactions. The exposed surface along the interface forms an elongated solvent-accessible groove of ~40 Å in length and ~10 Å in width, where the hydrolyzed products of *Ab*-54149 EPS were bound as described below. The central triangular channel created by the three β-helices is hydrophilic and accommodates many solvent molecules. Each wall of the channel contains two asparagine ladders, i.e. Asn343/Asn366 and Asn369/Asn397/Asn421.

Next to the RBD there are two triangular β-prisms, TP1 and TP2, connected by a highly interdigitated segment. In TP1, three four-stranded β-sheets from the three subunits assemble into the first β-prism with an exclusively hydrophobic interior, reminiscent of the β-prism in P22 TSP structure^20^. The triangular interior of TP1 is filled with regular stacks of aliphatic side chains: V502/I507/M520/F525 at the center and V500/I509/M518/T527 at each corner (Fig. 4B). Presumably these stacks stabilize the structure of TP1. Adjacent to TP1, the three polypeptide chains swap twice with the neighboring counterparts around the threefold axis and then assemble into the second twisted β-prism TP2. The interdigitated region and TP2 are stabilized by van der Waals association of side chains in the interior: I532/K547 at the center and L545/R549/Y566/R571/A588 at each corner (Fig. 4C), plus several H-bonds between Glu534, Lys547, Arg549, Asp564 and Arg571. In fact, the interdigitated segment is a 6-residue β-strand, and in a twisted manner this strand bridges the C- and N-terminal β-strands of TP1 and TP2, each from a different subunit in the trimer (Fig. 4D). As a consequence, the β-strands on each side of the β-prisms are indeed merged into a mixed, nine-stranded, strongly twisted β-sheet. Notably, ~44% of intersubunit interactions are concentrated in this region, despite the involvement of only ~19% residues, suggesting a critical role in maintaining the trimer structure. In the CTD, three β-sandwiches pack together to form a dome-like structure via the loop between the fourth and fifth β-strands in each β-sandwich (Fig. 3B,C). This loop slightly projects into the neighboring subunit and makes tight contacts with the fifth β-strand and the α-helix. The interface between the β-sandwiches is mainly stabilized by side chain H-bonds.

### Structure of the carbohydrate-binding groove

**Figure 5:**
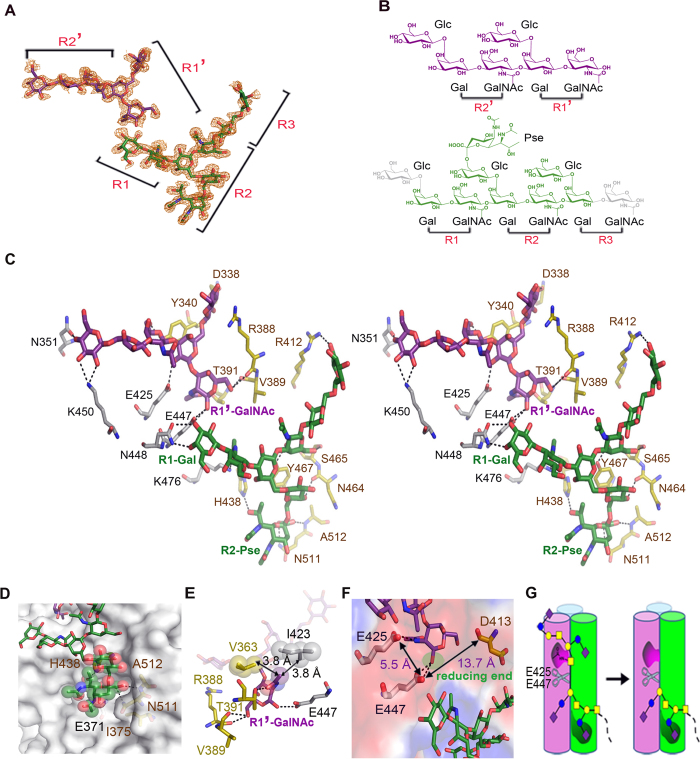
Substrate binding. (**A**) The 1σ *2F*_*o*_*-F*_*c*_ electron density map around the bound oligosaccharides is overlaid with the refined model, colored green for the R1-R2-R3 fragment and purple for R1′-R2′. **(B)** Chemical structure of the bound oligosaccharides. In this diagram, the unobserved R1-Glc*p* and R3-GalNAc*p* residues are colored gray. R2-Glc*p* is linked to a Pse. **(C)** Stereo view of the interactions between ΦAB6 TSP and the bound oligosaccharides. The fragments R1-R2-R3 and R1′-R2′ are colored as in **(B)**. Direct hydrogen bonds contributed by two different subunits (colored yellow and gray) are drawn with dotted lines. **(D)** The interaction of R2-Pse (spheres) and the enzyme (surface) is viewed close-up. **(E)** R1′-GalNAc*p* shows both hydrogen bonds and nonpolar interactions. **(F)** An acidic patch is formed by candidate residues for hydrolyzing the glycosidic bond of R1′-R1. **(G)** Schematic diagram for polysaccharide binding to the intersubunit groove of ΦAB6 TSP. In this diagram, E447 and E425 form the catalytic dyad. R2-Pse serves as the recognition site. The N-acetyl group of R1′-GalNAc*p* assists in positioning the anomeric carbon towards the nucleophilic residue Glu447. The representation of sugars is the same as in Fig. 2.

To obtain the structures of ΦAB6 TSP bound to different products, ΦAB6 TSP∆N crystals were soaked in reservoir solution containing the whole extract of *Ab*-54149 EPS for 2, 5, 8, 12, 15, 18, and 24 hours. Finally, structures obtained at the soaking times of 12 and 15 hours, but not others, showed 14 and 8 sugar residues corresponding to 5 and 3 repeat units, respectively (Figs 3B and 5A,B and Supplementary Fig. 11). In both structures, the bound oligosaccharides lie in an elongated groove near the interface of two adjacent β-helices, reminiscent of the intersubunit carbohydrate-binding site in Sf6 TSP^15^. No significant conformational change was found in comparison with the free-form structure (r.m.s. deviation = 0.068 Å and 0.055 Å, respectively, for all Cα atoms), indicating a rigid architecture of the enzyme. In the structure with 5-repeats, the oligosaccharide spans the groove from rung 4 of the β-helix to the TP1 of a neighboring subunit. The density for one Pse was observed (Figs 3B and 5A). Unexpectedly, the GalNAc*p*-(1 → 3)-Gal*p* glycosidic bond between the second and third repeat of the oligosaccharides indeed broke, resulting in two separate fragments in the binding groove, denoted R1-R2-R3 and R1′-R2′ (R: repeat unit) (Fig. 5A,B). The retention of stereochemistry at the reducing end (R1′-GalNAc*p*) indicated a retaining hydrolysis mechanism of ΦAB6 TSP^21^.

The ΦAB6 TSP-carbohydrate interaction is primarily mediated by H-bonds. Twelve direct H-bonds from the R1′-R2′ fragment to the residues Asn351, Glu425, Glu447 and Lys450 in one subunit and Asp338, Tyr340, Arg388, Val389, and Thr391 in a neighboring subunit stabilize R1′-R2′ in the binding groove (Fig. 5C). In addition, there are 8 water-mediated H-bonds between R1′-R2′ and the enzyme (Supplementary Fig. 12A). Towards the N-terminus, the groove is restricted by the T2 α-helix of rung 2, resulting in a ~70° bend between R1′ and R2′ (Figs 3B and 5A). As a consequence, R1′ is parallel to the longitudinal axis while R2′ is nearly perpendicular. The non-reducing end of R1′-R2′ is completely outward oriented (Fig. 5A), suggesting that fragments beyond R2′ would not contact the enzyme. Likewise, the backbone of R1-R2-R3 fragment adopts an L-shaped conformation stabilized by 11 direct H-bonds to the residues Glu447, Asn448 and Lys476 in one subunit and Arg412, His438, Asn464, Ser465, Tyr467, Asn511 and Ala512 in a neighboring subunit (Fig. 5C). Besides, there are 12 water-mediated H-bonds between R1-R2-R3 and enzyme (Supplementary Fig. 12A). Notably, three CH/π interactions were observed^22^, two from R1′-R2′ to the residues Tyr340 and Tyr374 (Supplementary Fig. 12B,C) and one from R1-R2-R3 to Tyr467 (Supplementary Fig. 12D). Presumably the H-bond network and the CH/π interactions confer the EPS-binding specificity of ΦAB6 TSP.

The densities for R1-Glc*p* and R3-GalNAc*p* were not visible, most likely due to their outward locations. The other Glc*p* residues are also outwardly oriented, except for R2-Glc*p*. The Pse connects to R2-Glc*p* and fits well in a wide pocket made by Glu371, Ile375, His438, Asn511 and Ala512 from two different subunits (Fig. 5D). Three H-bonds to His438, Asn511, and Ala512 also stabilize the Pse in the pocket (Fig. 5C), implying that this Pse serves as a recognition site for ΦAB6 TSP. In addition, the N-acetyl group of R1′-GalNAc*p* is inserted into a small cavity stabilized by two H-bonds to Thr391 and Glu425 from different subunits (Fig. 5E). The methyl moiety of this N-acetyl group is in further contact with Val363 and Ile423 (Fig. 5E). Consequently, this orientation allows the anomeric carbon of R1′-GalNAc*p* to be in the proximity of Glu447 (Fig. 5F), a possible nucleophilic residue as described below.

The R1′-R2′ fragment was released in the structure obtained at the 15-hour soaking time, leaving the R1-R2-R3 fragment in the binding groove (Supplementary Fig. 11), suggesting that this structure represents the post cleavage stage. Interestingly, the release of the R1′-R2′ fragment is consistent with the observation of the digested products as revealed by MS spectra (Supplementary Fig. 9), implying that R1′-R2′ represents a terminal fragment of the polysaccharides (Fig. 5G).

### Catalytic center

It has been known that retaining glycoside hydrolases operate through a two-step mechanism by utilizing two carboxylate residues, one acting as a nucleophile and the other as an acid/base^21^. In the complex crystal structure, the distance between the anomeric carbon of R1′-GalNAc*p* and the side chain of Glu447 is only 3.3 Å, implying that Glu447 is the nucleophilic residue during catalysis. In fact, R1′-GalNAc*p* lies on a prominent acidic surface patch made up of the residues Glu425 and Glu447 in one subunit and Asp413 in a neighboring subunit (Fig. 5F). The distance from Glu447 to Glu425 is 5.5 Å; to Asp413, 13.7 Å (Fig. 5F). Considering the typical distance from the catalytic nucleophile to the general acid/base in a retaining glycoside hydrolase is between 4.5 and 5.5 Å 23, Glu425 is a prime candidate to be the acid/base. To verify their catalytic roles, Asp413, Glu425 and Glu447 were individually mutated to their respective amide residues. Mutations at Glu425 and Glu447 drastically reduced the turnover rate of the enzyme with little effect on the binding affinity (Table 2 and Supplementary Fig. 13), whereas mutation at Asp413 only slightly decreased the enzyme activity. Consistent results were also obtained when spotting these mutant enzymes on a top agar inoculated with *Ab*-54149 (Fig. 1A). These results confirm that Glu425 and Glu447 make substantial contributions to catalysis. Consequently, the catalytic carboxylates of ΦAB6 TSP reside in the same subunit, unlike those of Sf6 TSP found in two different subunits.

## Discussion

*Acinetobacter baumannii* has captured significant attention recently in clinical and epidemic researches owing to a significant increase of its antibiotic-resistant strains. Using bacterial exopolysaccharide (EPS) to generate glycovaccines is one of the new treatments to combat bacterial infections, which have been applied to reduce the spreads of *Haemophilus influenza type b, Streptococcus pneumonia*, and *Neisseria meningitides*^24^. By conjugating polysaccharide to carrier protein, glycoconjugate vaccine (GCV) can elicit a strong and long-lasting immune response compared to whole EPS^11^, making GCV useful in combating antibiotic-resistant bacteria^25^,^26^. A critical problem to GCV development is heterogeneity. It may be overcome by obtaining proper carbohydrate repeat units, which can induce stronger immune responses than whole bacterial EPS^27^. Because chemical synthesis of polysaccharide can be time-consuming with low yields^28^ and chemical cleavage for bacterial EPS usually turns out a mixture of different sizes^29^, enzymatic digestion may provide an alternative. In this regard, bacteriophage TSPs can be employed to produce more homogenous fragments of bacterial EPS. The homogeneity and preferred size of oligosaccharides may be achieved through protein engineering.

In the present study, the TSP from the bacteriophage ΦAB6 (ΦAB6 TSP), which specifically hydrolyzed the EPS of *A. baumannii* strain 54149 (*Ab*-54149), was characterized. The whole extract and the ΦAB6 TSP-digested products of *Ab*-54149 EPS were also analyzed by NMR and LC-ESI-MS. Recently, the chemical structures of EPS of two antibiotic-resistant *A. baumannii* strains have been reported; one is aminoglycoside-resistant and another carbapenem-resistant^30^,^31^. Both polysaccharides indeed have the same structure. Interestingly, the structure of *Ab*-54149 EPS characterized here is the same as the two antibiotic-resistant *A. baumannii* strains, implying that substrates produced using the TSP may be useful for a GCV.

The structure of the N-terminally-truncated ΦAB6 TSP determined here revealed a trimeric β-helix architecture. The structure exhibits an organization similar to the structures of other *Podoviridae* TSPs, despite the lack of homology in amino acid sequence (Supplementary Fig. 14). A close comparison of these TSP structures revealed ΦAB6 TSP to be more elongated in both the monomer and trimer, likely due to the extensive structure of triangular β-prisms as well as the fewer coils and smaller loop insertions in the β-helix domain (Supplementary Fig. 10). In contrast to the β-helix of other TSPs, which typically contains 13 rungs, the β-helix in ΦAB6 TSP has only 8 rungs, and 4 of them contains the fourth β-strand never before seen in other TSPs. TP1 of ΦAB6 TSP shares the structural feature of the C-terminal intertwined region of P22 TSP, i.e. with regular aliphatic side chain stacks in the center and corners of the triangular inner space. TP2 is unique in the TSP family, as it is more twisted and stabilized by mixed aliphatic/polar side chain stacks and side chain H-bonds. The N-terminal particle-binding domain of ΦAB6 TSP is dominated by a β-stranded scaffold, similar to the N-terminal domain of P22 TSP, but the β-sheets in these two structures are nearly perpendicular to each other. By comparison, the N-terminal domain of ΦAB6 TSP is significantly different from those of Sf6 TSP and HK620 TSP, which are organized into α-helical bundles (Supplementary Fig. 10). In addition, the C-terminal β-sandwich domain of ΦAB6 TSP resembles the C-terminal domain of Sf6 TSP and HK620 TSP, but the orientations of the β-sandwiches are quite different in these structures. As a consequence, the buried surfaces between the β-sandwiches are also different.

The structures of ΦAB6 TSP in complexes with the products revealed the intersubunit carbohydrate-binding grooves. To date, although more than 30 right-handed β-helix structures have been reported^15^, the intersubunit carbohydrate-binding site can only be identified in the structures of SF6 TSP and an inulin fructotransferase from *Bacillus sp. snu-7*^32^. Notably, the inulin fructotransferase depolymerizes inulin by successively removing the terminal difructosaccharide units^32^. In the complex structure of ΦAB6 TSP, the N-terminal side of the carbohydrate-binding groove is closed by the T2 α-helix of rung 2, and the distance between the reducing end of R1′-R2′ fragment and the α-helix is ~17 Å. Because the backbone of a 3-repeat unit fragment is more than 22 Å in length, the binding groove in this N-terminal area could only accommodate 2 repeat units. This finding is also in agreement with the sizes of digested products as mentioned above. In this regard, this area of carbohydrate-binding groove represents a target for rational design and engineering of the enzyme to produce desired products. Indeed, in P22 TSP and HK620 TSP, the size of digested product also reflects the dimension of this N-terminal area of carbohydrate-binding groove^16^,^33^. Conceivably, these TSPs might first bind to the terminal fragments of bacterial surface polysaccharides and catalyze their successive removal to gain access to the host cell.

The branched Glc*p* residues in R1′-R2′ and R1-R2-R3 are outwardly oriented and flexible in the complex structures, with the exception of R2-Glc*p*. As a consequence, the densities for the Pse residues were not visible, except for the one connected to R2-Glc*p*, indicating that most Pse residues are not involved in the binding to ΦAB6 TSP. By contrast, the Pse at R2-Glc*p* fits well to a pocket in the binding groove, suggesting a recognition site in the substrate for ΦAB6 TSP (Fig. 5G). This is reminiscent of the recognition site in *Salmonella* O-antigens for P22 TSP, which relies on a branched sugar residue as well^34^,^35^. It is notable that we did not observe the degradation products when the extract of *Ab*-SK44 EPS, which contains β-GalNAc*p*-(1 → 3)-β-Gal*p* linkages but no Pse, was treated with ΦAB6 TSP (data not shown)^36^. This also supports the recognition role of the Pse residue. On the other hand, the stoichiometric Pse in the main digestion product of *Ab*-54149 EPS correlates very well with the high Pse content of the polysaccharide, as estimated by using 1D NMR spectra (Supplementary Fig. 15).

Regarding the catalysis mechanism, mutations at the catalytic carboxylate residues of P22 TSP and Sf6 TSP to their respective amide reduced the enzyme activity to less than 0.1%^15^,^34^. By contrast, ~1.6% and ~1.9% activity were still detectable for the mutants E425Q and E447Q of ΦAB6 TSP, respectively (Table 2), implying that an alternative but minor hydrolysis mechanism might coexist. By comparing the chemical structure of substrate of ΦAB6 TSP with those of P22 TSP and Sf6 TSP, it is reasonable to assume that the N-acetyl group next to the anomeric carbon can act as an intramolecular nucleophile to proceed with the reaction. Indeed, this is common for certain N-acetylhexosaminidases^37^.

In conclusion, a new bacteriophage tailspike protein that specifically hydrolyzed the EPS of *Ab*-54149 was characterized. *Ab*-54149 EPS exhibited the same chemical structure as two other antibiotic-resistant *A. baumannii* strains. The structures of ΦAB6 TSP in complexes with the semi-hydrolyzed products provide deep insights into the substrate recognition and product specificity of the enzyme and also elucidate a retaining hydrolysis mechanism, for which the catalytic residues have been verified by site-directed mutagenesis. These results constitute a structural basis for engineering the enzyme to produce desired oligosaccharides, which can be useful for the development of GCVs against *A. baumannii* infections.

## Methods

### Protein expression and purification

The DNA fragments encoding the amino acid sequences 1–699 (ΦAB6 TSP) and 136–699 (ΦAB6 TSP∆N) of the ΦAB6 tailspike protein, respectively, were amplified from the phage genomic DNA and inserted into the vector pET28a (Novagen) via NdeI and XhoI cloning sites. After the sequences were confirmed, the vectors were transformed into the *E. coli* BL21(DE3) (Novagen). The cells were grown in Luria-Bertani (LB) medium supplemented with 50 μg/mL kanamycin at 37 °C until the cell density reached OD600 of 0.4–0.6. The cultured cells were induced with 0.1 mM IPTG at 20 °C overnight, and the cells were harvested by centrifugation (6,000 rpm) at 4 °C for 30 min and resuspended in buffer A (25 mM Tris-HCl and 100 mM NaCl, pH 7.5). The cells were lysed by passing through a French Press (Constant System Ltd, Constant System TS 2.2kw) three times and the lysate was clarified by centrifugation (20,000 rpm) at 4 °C for 60 min. The supernatant was loaded onto an open column filled with nickel-charged chelating resin (Qiagen) and pre-equilibrated with buffer A. The recombinant protein was eluted with 100–300 mM imidazole and the eluted fractions were pooled and then dialyzed against buffer A at 4 °C overnight. The recombinant proteins were further purified by a Superdex-200 gel-filtration column (GE-Healthcare), leading to near homogeneity. The protein was concentrated to ~10 mg/mL by using a 30 K cut-off centrifuge filter (Millipore).

The expression vectors for the mutants D413N, E425Q, and E447Q were constructed using the QuikChange II site-directed mutagenesis Kit (Stratagene) following the manufacturer′s instructions. The protein expression and purification procedures were the same as described above. The Se-labeled ΦAB6 TSP∆N was prepared on the basis of a nonauxotrophic protocol using the commercially available Se-Met medium (Molecular Dimensions)^38^.

### Extraction of bacterial surface polysaccharides

The crude extracts of bacterial surface polysaccharides were obtained on the basis of protocol reported by Zamze *et al* with several modifications^39^. Briefly, the bacterial cells were cultured with LB medium (for *A. baumannii*) or grown on LB plate (for *K. pneumoniae*) at 37 °C for 15 h and the cultured cells were collected. The cells were suspended in d.d. water and heated to 100 °C for 20 min to lyse the cells. The cell lysate was clarified by centrifugation at 10,000 rpm for 20 min, and the supernatant containing the bacterial surface polysaccharides was incubated with 80% acetone overnight to precipitate the polysaccharides. The precipitate dissolved in 10 mM Tris-HCl and 1 mM CaCl_2,_ pH 7.5, was treated with ribonuclease (Sigma) and deoxyribonuclease I (Roche) at 37 °C for 6 h and then treated with proteinase K (Bioshop) for 12 hr. Subsequently, the sample was dialyzed against d.d. water by using a 1 kDa-cutoff membrane and then lyophilized. Finally, the crude polysaccharide extracts were further purified by a HW-65F gel-permeation column (TSK-GEL) to remove the contamination of bacterial organisms. The presence and concentration of the extracted polysaccharides were determined by the phenol-sulfuric acid method^40^.

### Digestion of *A. baumannii* surface polysaccharide by ΦAB6 TSP

Twenty mg crude extract of *Ab*-54149 surface polysaccharide dissolved in 25 mM Tris-HCl and 100 mM NaCl, pH 7.5 was incubated with 500 μg of purified ΦAB6 TSP at 37 °C for 6 h, and then the digestion reaction was terminated by heating to 100 °C for 15 min. The denatured proteins were removed by centrifugation. Subsequently, the digested products were loaded onto a P-6 column (Bio-Rad) and the oligosaccharides were eluted with d.d. water. The eluted fractions were pooled and lyophilized for subsequent enzyme activity and chemical structure analyses.

### Top agar assay

The top agar assay was performed according to the protocol reported previously^41^. Briefly, the LB agar in a petri dish was overlaid with 10 mL top agar pre-inoculated with the fresh culture of *Ab*-54149. After the top agar was solidified, 3 μL (~1 μg/μL) of either the wide-type, truncated, or mutant ΦAB6 TSP were spotted on the petri dish and incubated overnight at 37 °C. The enzyme activity was evaluated by measuring the presence and dimension of translucent halos on the surface of the agar.

### Structure determination of *Ab*-54149 surface polysaccharide by NMR

The digested products or whole polysaccharide of *Ab*-54149 surface polysaccharide were dissolved in 99.95% D_2_O. NMR experiments were performed using Bruker Avance 500 MHz spectrometer equipped with a cryoprobe, and the spectra were acquired at 298 K. 1D and 2D spectra were obtained using standard Bruker software, and Bruker TopSpin 2.1 program was used to process the NMR data. All two-dimensional homo and heteronuclear experiments (correlation spectroscopy, COSY; Overhauser effect spectroscopy, NOESY; heteronuclear multiple quantum coherence, HSQC; heteronuclear multiple bond correlation, HMBC) were carried out with the standard pulse sequences provided by Bruker. The mixing time for One-dimensional TOCSY experiment is 120 ms. The NOESY spectra were recorded at mixing time at 60 ms in order to identify genuine NOE effects. For homonuclear experiment, 256 FIDS of 2048 complex data point were collected with 20 scans per FID. For HSQC and HMBC spectra, 256 FIDS of 2048 complex data point were collected with 32 scans per FID, respectively. The assignment of protons chemical shifts were achieved by COSY, NOESY and One-dimensional TOCSY. In addition, the assignment of carbon chemical shifts was performed by HSQC and HMBC.

### Mass spectrometry analysis of digested products of *Ab*-54149 exopolysaccharide

LC**-**ESI-MS and LC-ESI-MS-MS analyses were done on a LTQ Orbitrap XL ETD mass spectrometer (Thermo Fisher Scientific, San Jose, CA) equipped with standard ESI ion source. 5 μL of sample was injected at a flow rate of 50 μL/min in 80% ACN/H_2_O with 0.1% FA by Ultimate 3000 RSLC system from Dionex (Dionex Corporation, Sunnyvale, CA). The conditions for full-scan MS are as follows: mass range m/z 0-6000 and resolution 60,000 at m/z 400. The target ions were sequentially isolated for MS2 by LTQ. Electrospray voltage was maintained at 4 kV and capillary temperature was set at 275 °C.

### Enzyme kinetic assay

The hydrolytic activity of ΦAB6 TSP towards polysaccharides was evaluated by quantifying the production of reducing end with the reagent 3,5-dinitrosalicylic acid (DNS), as described previously^42^. For common activity assay, the extract of Ab-54149 surface polysaccharide dissolved in 20 mM HEPES/MES/sodium acetate, pH 5.0, was treated with the purified ΦAB6 TSP at 37 °C. The final concentration of the polysaccharides was 5 mg/mL. The reaction was quenched at different time intervals by heating to 100 °C for 15 min, and then the denatured enzyme was removed by centrifugation. Subsequently, an aliquot of the digested products was mixed with an equal volume of DNS reagent (20 mg/mL) in 0.7 M NaOH, and the mixture was heated to 100 °C for 5 min. The hydrolytic activity of the enzyme was evaluated by measuring the absorption at 535 nm using an UV spectrophotometry. For kinetic study of the enzyme, the concentrations of polysaccharides were in the range of 2–60 mg/mL. The kinetic parameters were obtained by fitting the data of initial velocities versus polysaccharide concentrations with the Michaelis–Menten equation.

### Crystallization and X-ray data collection

We failed to obtain crystals using the full-length ΦAB6 TSP or using ΦAB6 TSP∆N in Tris-HCl buffer, so we tried to grow the crystals of ΦAB6 TSP∆N in a different protein buffer. The purified ΦAB6 TSP∆N was dialyzed against 25 mM HEPES and 100 mM NaCl, pH 7.5 and then concentrated to ~10 mg/mL. The initial crystallization screening of ~1,000 conditions was accomplished in the Core Facilities for Protein Structural Analysis (CFPSA), Academia Sinica (Taipei, Taiwan). The resulting initial conditions were further refined manually. Finally, two crystallization conditions were selected, i.e., (i) 1.0 M sodium malonate, pH 7.0 and (ii) 0.9 M sodium citrate and 0.1 M sodium cacodylate, pH 6.5. The crystals were grown at 20 °C by mixing the ΦAB6 TSP∆N solution with equal volume of crystallization buffers via the hanging-drop vapor-diffusion method. The rhombus-shaped crystals with dimensions reaching 0.15 × 0.15 × 0.2 mm appeared within two days. The crystals for product-bound forms of ΦAB6 TSP∆N were obtained by soaking the free-form crystals into reservoir solution containing 5 mM surface polysaccharide of *Ab*-54149 at 20 °C for various periods between 1 and 24 hours. The crystals for Se-labeled ΦAB6 TSP∆N could only be obtained by using the second crystallization buffer. The single-wavelength anomalous diffraction (SAD) data at 2.69-Å resolution for crystals of Se-labeled ΦAB6 TSP∆N was collected at the beamline 12B2 of SPring-8 (Hyogo, Japan). The high-resolution data and those for different product-bound forms were collected at the beamlines 15A1, 13B1, or 13C1 of National Synchrotron Radiation Research Center (Hsinchu, Taiwan). Before being mounted on the goniometer, the crystals were briefly immersed in reservoir solution containing 12% (v/v) glycerol as cryoprotectant. All diffraction data were processed and scaled with the *HKL-2000* package^43^. The data collection statistics are listed in Table 1. The space group of the crystals is *C*_2_ with the typical unit cell dimensions of *a* = 135.5 Å, *b* = 78.0 Å, *c* = 248.0 Å, *α* = 90.0°, *β* = 100.5°, and *γ* = 90.0°. The asymmetric unit comprises a ΦAB6 TSP∆N trimer with an estimated solvent content of 65.25%.

### Structure determination and refinement

The crystal structure of ΦAB6 TSP∆N was solved by the SAD-phasing method with the program *AutoSol* within the *PHENIX* software suite^44^ and using the 2.69-Å resolution data collected at the wavelength of absorption peak. The positions of the 30 Se sites, with occupancies between 0.63 and 1.0, for the ΦAB6 TSP∆N trimer in the asymmetry unit were determined. The Se sites were then refined and the initial phases were improved by density modification with *AutoSol*. Approximate 93% model was automatically traced into the Se-phased electron density map with the program *AutoBuild*, and the remainder was manually built with *Coot*^45^. The resulting model was subjected to computational refinement with the program *REFMAC5*^46^. Throughout refinement, a randomly selected 5% of the data was set aside as a free data set, and the model was refined against the remaining data with *F* > 0 as a working data set. The parameters for ideal protein geometry of Engh and Huber were used during refinement^47^. Subsequently, iterative rounds of model adjustment with *Coot* and refinement with *REFMAC5* were performed using the 1.48-Å resolution data set to improve the quality and completeness of the structure. The well-ordered malonate and water molecules were located with *Coot*. Finally, the refinement converged at a final *R* factor and *R*_free_ of 0.136 and 0.156, respectively, using anisotropic temperature factors. The stereochemical quality of the refined structure was checked with the program *PROCHECK*^48^. The final refinement statistics are listed in Table 1. With respect to the structures of product-bound ΦAB6 TSP∆N, the initial difference Fourier maps were obtained by using the refined structure of free-form ΦAB6 TSP∆N, and the subsequent refinements were the same as described above. The molecular figures were generated with *PyMOL* (Schrödinger, New York, USA).

### Analytical ultracentrifugation analysis

Ultracentrifugation sedimentation experiments were performed at 20,000 rpm in a Beckman XL-A analytical ultracentrifuge (Beckman Instruments, Fullerton, Calif, USA) equipped with the standard double-sector centerpieces and using an An-60 Ti rotor. The UV absorption at 280 nm was scanned every 4 min for 250 scans. The data were analyzed with the software SEDFIT. The protein sample was in 25 mM Tris-HCl and 300 mM NaCl, pH 8.0. The sample was visually checked for clarity after ultracentrifugation. No precipitation was observed.

## Additional Information

**How to cite this article**: Lee, I.-M. *et al*. Structural basis for fragmenting the exopolysaccharide of *Acintobacter baumannii* by bacteriophage ΦAB6 tailspike protein. *Sci. Rep.* **7**, 42711; doi: 10.1038/srep42711 (2017).

**Publisher's note:** Springer Nature remains neutral with regard to jurisdictional claims in published maps and institutional affiliations.

## Supplementary Material

Supplementary Information

## Figures and Tables

**Table 1:** Data-collection and refinement statistics.

	Se-ΦAB6 TSP	ΦAB6 TSP	ΦAB6 TSP -5 R.U.	ΦAB6 TSP -3 R.U.
**Data collection**				
Wavelength (Å)	0.979299 Å	1.0 Å	1.0 Å	1.0 Å
Resolution (Å)	30−2.69 (2.79−2.69)	20−1.48 (1.53−1.48)	20−1.48 (1.53−1.48)	30−1.89 (1.96−1.89)
Unit cell dimensions				
*a* (Å)	135.45	135.02	135.81	134.93
*b* (Å)	78.21	77.98	78.39	77.95
*c* (Å)	248.18	248.08	247.74	247.74
*α, β, γ* (°)	90.0, 100.5, 90.0	90.0, 100.5, 90.0	90.0, 100.5, 90.0	90.0, 100.5, 90.0
Total observations	356,683	2,847,907	1,121,692	1,031,426
Unique reflections	69,784 (6,408)	406,268 (40,050)	399,721 (41,740)	203,248 (20,345)
Multiplicity	5.1 (5.0)	7.0 (7.6)	2.8 (2.9)	5.1 (4.0)
Completeness (%)	98.5 (91.3)	96.6 (95.3)	94.1 (99.0)	99.8 (99.9)
*I*/σ(*I*)	43.7 (21.0)	34.3 (3.3)	20.8 (2.0)	18.4 (2.7)
*R*_merge_ (%)	8.0 (11.6)	5.4 (60.6)	5.4 (50.1)	13.1(79)
**Refinement**				
Resolution (Å)		19.98−1.48	19.96−1.48	24.89−1.89
Reflections [>0σ(*F*)], working/test		355,526/19,849	335,547/18,826	180,320/9,852
*R* factor/*R*_free_		0.136/0.156	0.136/0.164	0.122/0.162
R.m.s.d., bond lengths (Å)/ angles (°)		0.004/1.062	0.005/1.175	0.005/1.127
Average *B* factor (Å^2^)/No. of atoms				
Protein		14.61/12537	14.66/12579	20.88/12564
Sugar			28.08/537	48.11/318
Malonate		26.71/63	28.99/21	43.59/42
Water		32.63/2786	32.88/2883	38.85/2138
Ramachandran plot, residues in (%)				
Most favored regions		88.0	88.2	88.4
Additionally allowed regions		11.0	10.8	10.6
Generously allowed regions		0.6	0.6	0.6
Disallowed regions		0.4	0.4	0.4
PDB code		5JS4	5JSD	5JSE

These crystals belong to the space group *C2* and one asymmetric unit comprises one ΦAB6 TSP trimer. Values in parentheses correspond to the highest resolution shell.

R.U. is repeat units of *A. baumannii* exopolysaccharide.

**Table 2:** Kinetic data of wild-type and mutant ΦAB6 TSPs.

		*k*_cat_ (min^−1^)	*K*_m_ (mg • ml^−1^)	*k*_cat_/*K*_m_ (ml • mg^−1^ • min^−1^)	Relative activity (%)
**ΦAB6 TSP**	WT	244 ± 14	3.79 ± 0.77	64.38 ± 18.1	100
	E425Q	11.8 ± 1.85	10.9 ± 4.7	1.08 ± 0.39	1.6
	E447Q	9.76 ± 1.43	7.89 ± 3.56	1.23 ± 0.4	1.9
	D413N	220 ± 12.5	3.9 ± 0.9	56.4 ± 13.8	87.6
