# Supplementary material for: Structural basis for fragmenting the exopolysaccharide of Acinetobacter baumannii by bacteriophage ΦAB6 tailspike protein
Source: Sci Rep. 2017 Feb 17;7:42711. doi: 10.1038/srep42711 (PMC5314372; doi:10.1038/srep42711)
Supplement: Supplementary Information [file srep42711-s1.doc]

**Supplementary Information**

Structural basis for fragmenting the exopolysaccharide of *Acinetobacter baumannii* by bacteriophage ΦAB6 tailspike protein

I-Ming Lee^1,2^, I-Fan Tu^2^, Feng-Ling Yang^2^, Tzu-Ping Ko^2,5^, Jiahn-Haur Liao^2^, Nien-Tsung Lin^3^, Chung-Yi Wu^4^, Chien-Tai Ren^4^, Andrew H.-J. Wang^2,5^, Ching-Ming, Chang^2^, Kai-Fa Huang*^,2,5^ and Shih-Hsiung Wu*^,1,2,6^

^1^Institute of Biochemical Sciences, National Taiwan University, Taipei 106, Taiwan

^2^Institute of Biological Chemistry, Academia Sinica, Taipei 115, Taiwan

^3^ Master program in Microbiology and Immunology, Tzu Chi University, Hualien 970, Taiwan

^4^Genomics Research Center, Academia Sinica, Taipei 115, Taiwan

^5^Core Facilities for Protein Structural Analysis (CFPSA), Academia Sinica, Taipei 115, Taiwan

^6^Department of Chemistry, National Taiwan University, Taipei 106, Taiwan


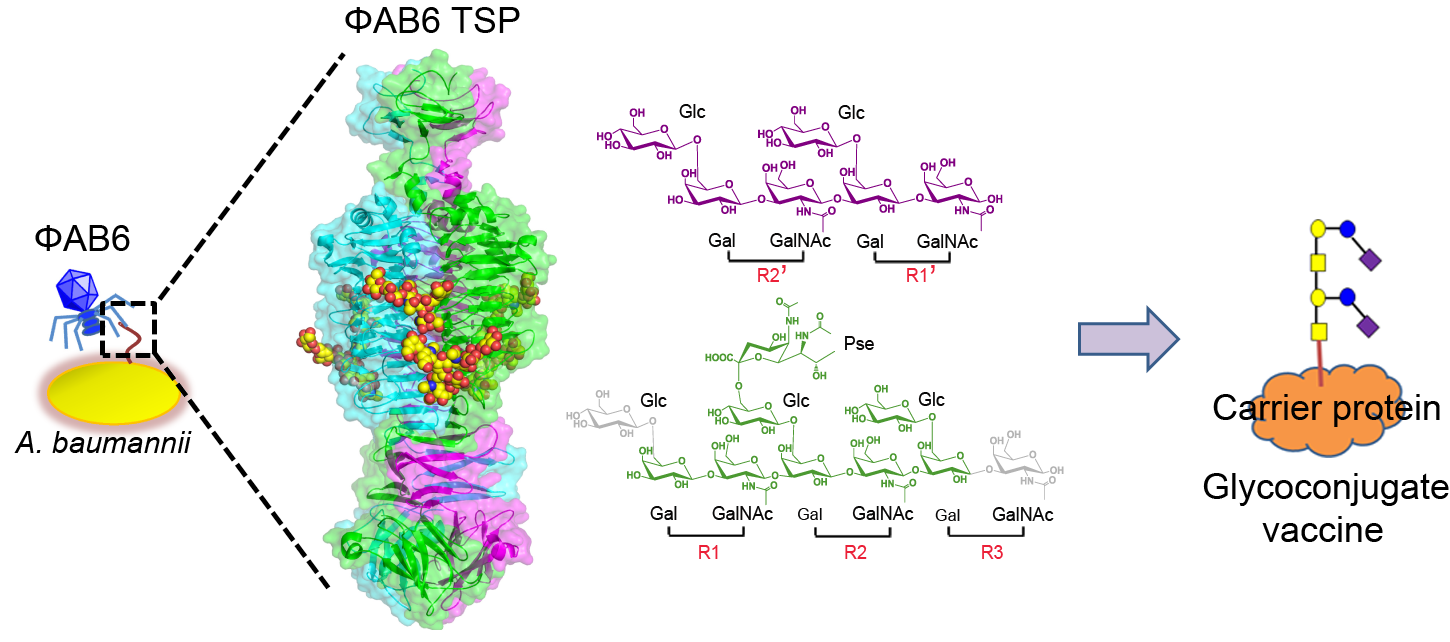
**Supplementary Figures**

**Supplementary Figure 1. Illustration of glycoconjugate vaccine production by using bacteriophage tailspike protein (TSP).** To combat pathogenic antibiotic-resistant bacteria, a promising strategy is using TSP-digested fragments of bacterial exopolysaccharide to make glycoconjugate vaccine.

**
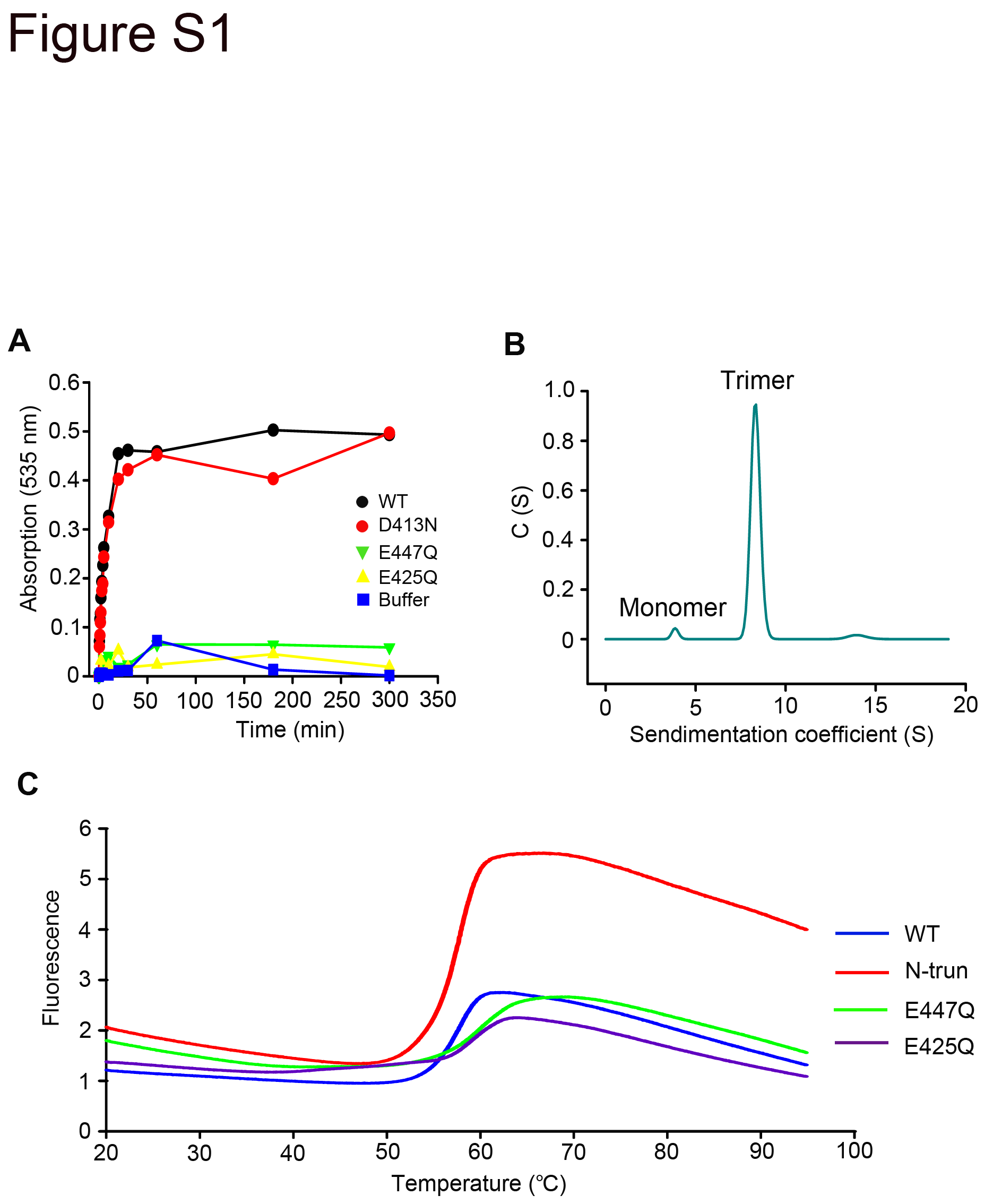
**

**Supplementary Figure 2. Characterization of recombinant ΦAB6 TSP.** (A) Time-dependent depolymerization of *Ab*-54149 exopolysaccharide (10 mg/mL) catalyzed by wild-type and mutant ΦAB6 TSP. The enzyme activity was evaluated by quantifying the production of reducing end using the reagent 3,5-dinitrosalicylic acid (DNS). (B) Analytical ultracentrifugation analysis of ΦAB6 TSP. According to the molecular size calculated from the sendimentation coefficients, the major form of ΦAB6 TSP in solution is a trimer. (C) Comparison of the thermal stabilities of wild-type, N-terminally truncated, and mutant ΦAB6 TSP analyzed by the SYPRO Orange dye (Invitrogen).


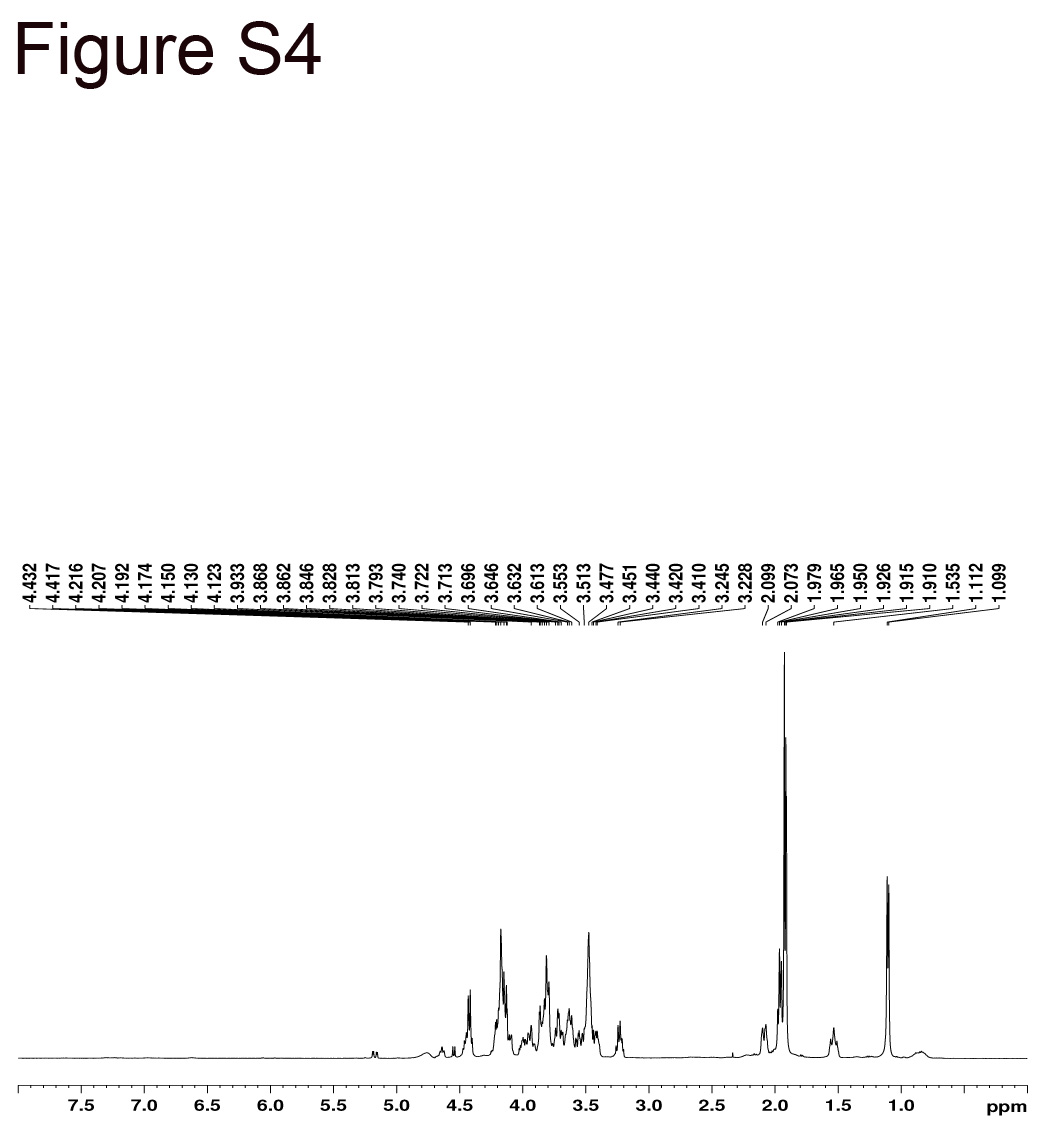
**Supplementary Figure 3. ^1^H NMR (500 MH_Z_, D_2_O) analysis of ΦAB6 TSP-digested products of *Ab*-54149 exopolysaccharide.**


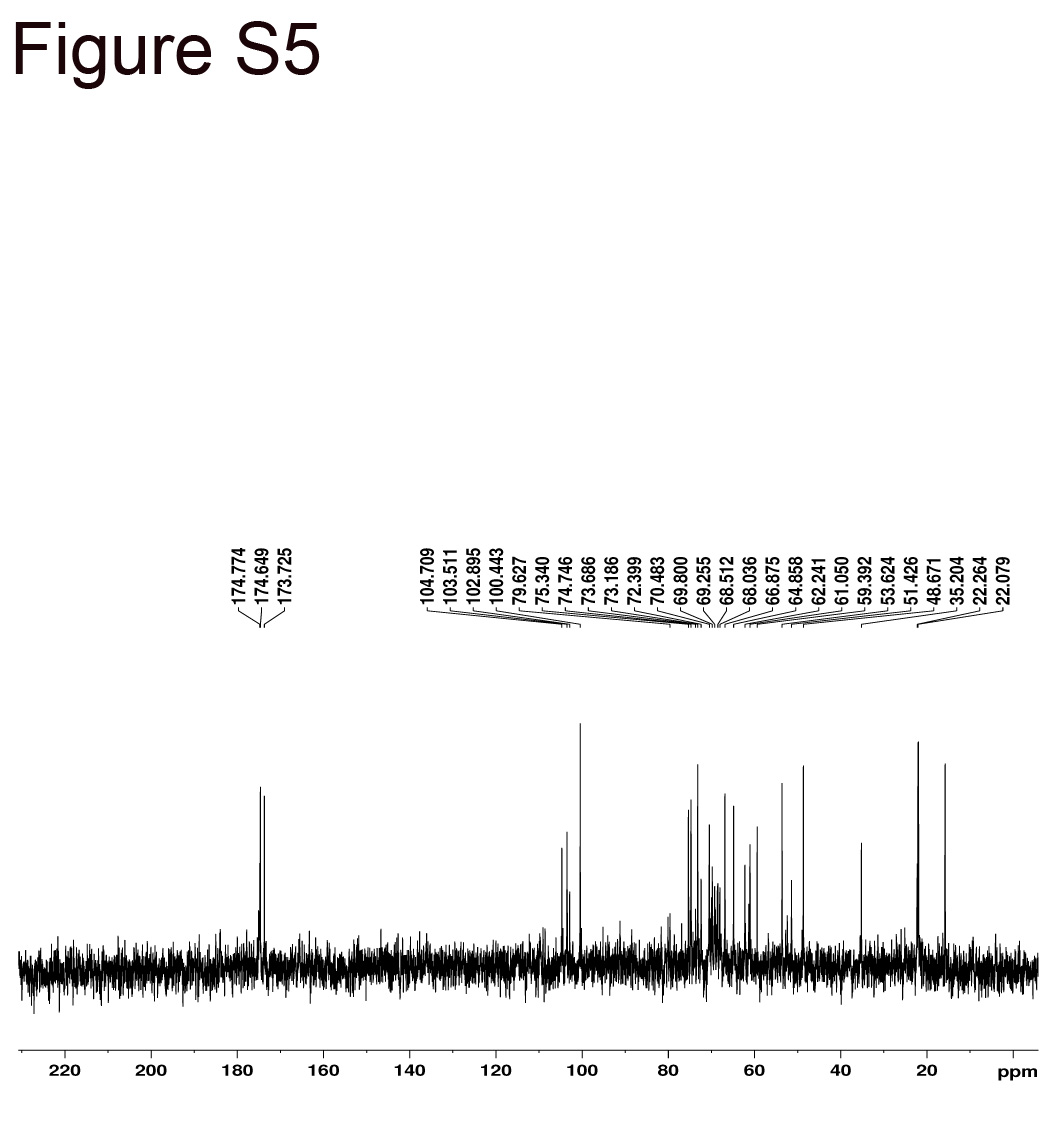


**Supplementary Figure 4.** **^13^C NMR (500 MH_Z_, D_2_O) analysis of ΦAB6 TSP-digested products of *Ab*-54149 exopolysaccharide.**


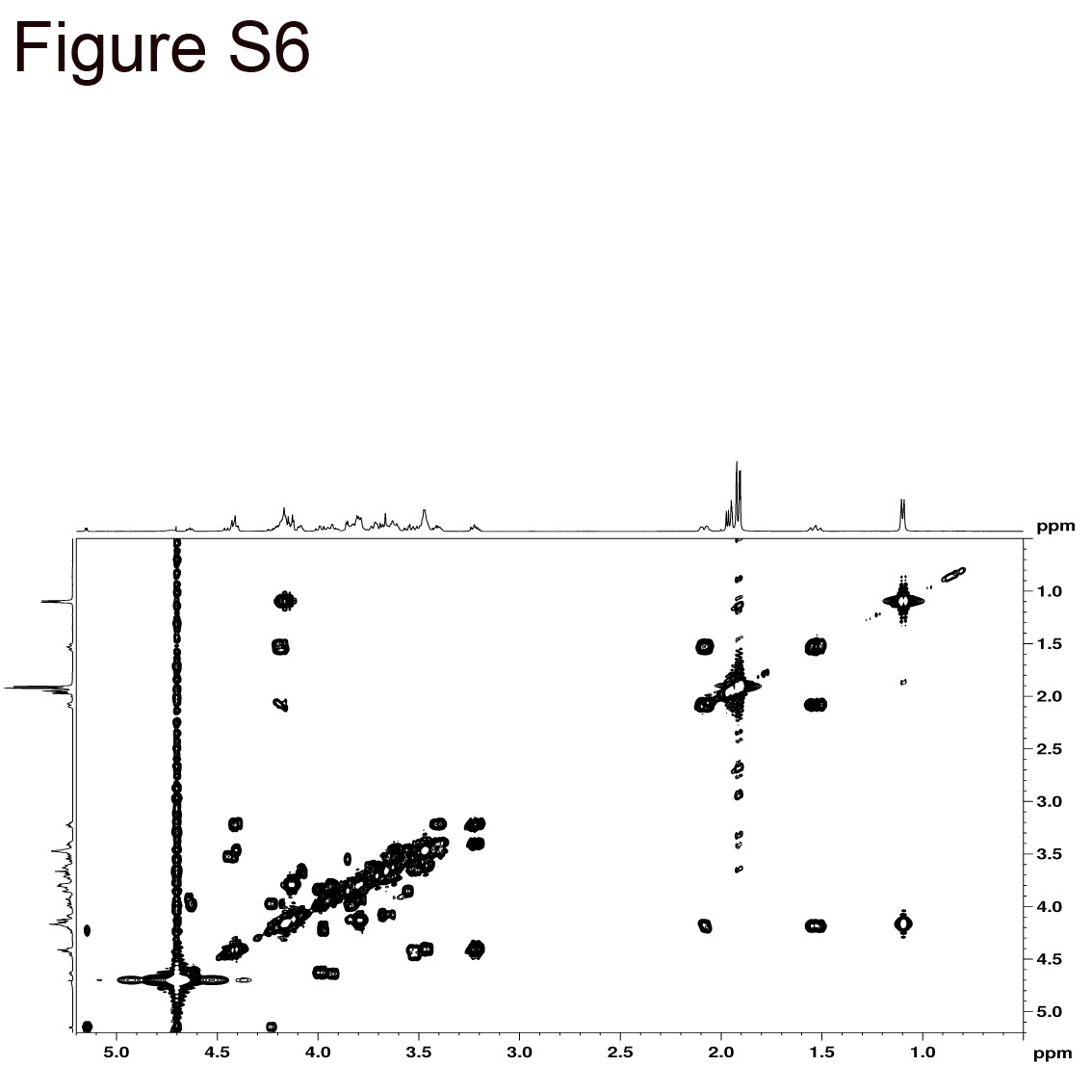
**Supplementary Figure 5. COSY spectrum (500 MH_Z_, D_2_O) of ΦAB6 TSP-digested products of *Ab*-54149 exopolysaccharide.**


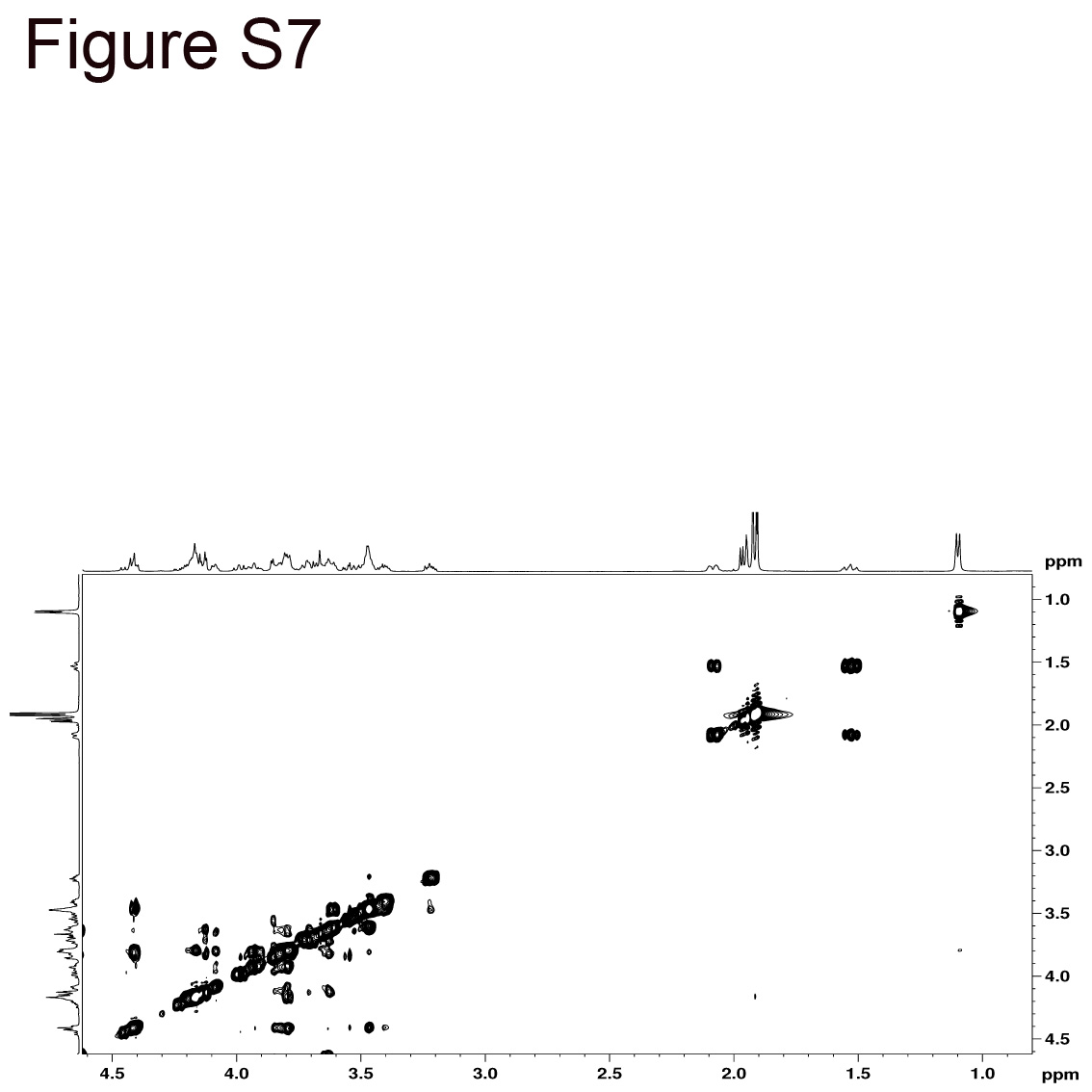


**Supplementary Figure 6. NOESY spectrum (500 MH_Z_, D_2_O) of ΦAB6 TSP-digested products of *Ab*-54149 exopolysaccharide.**


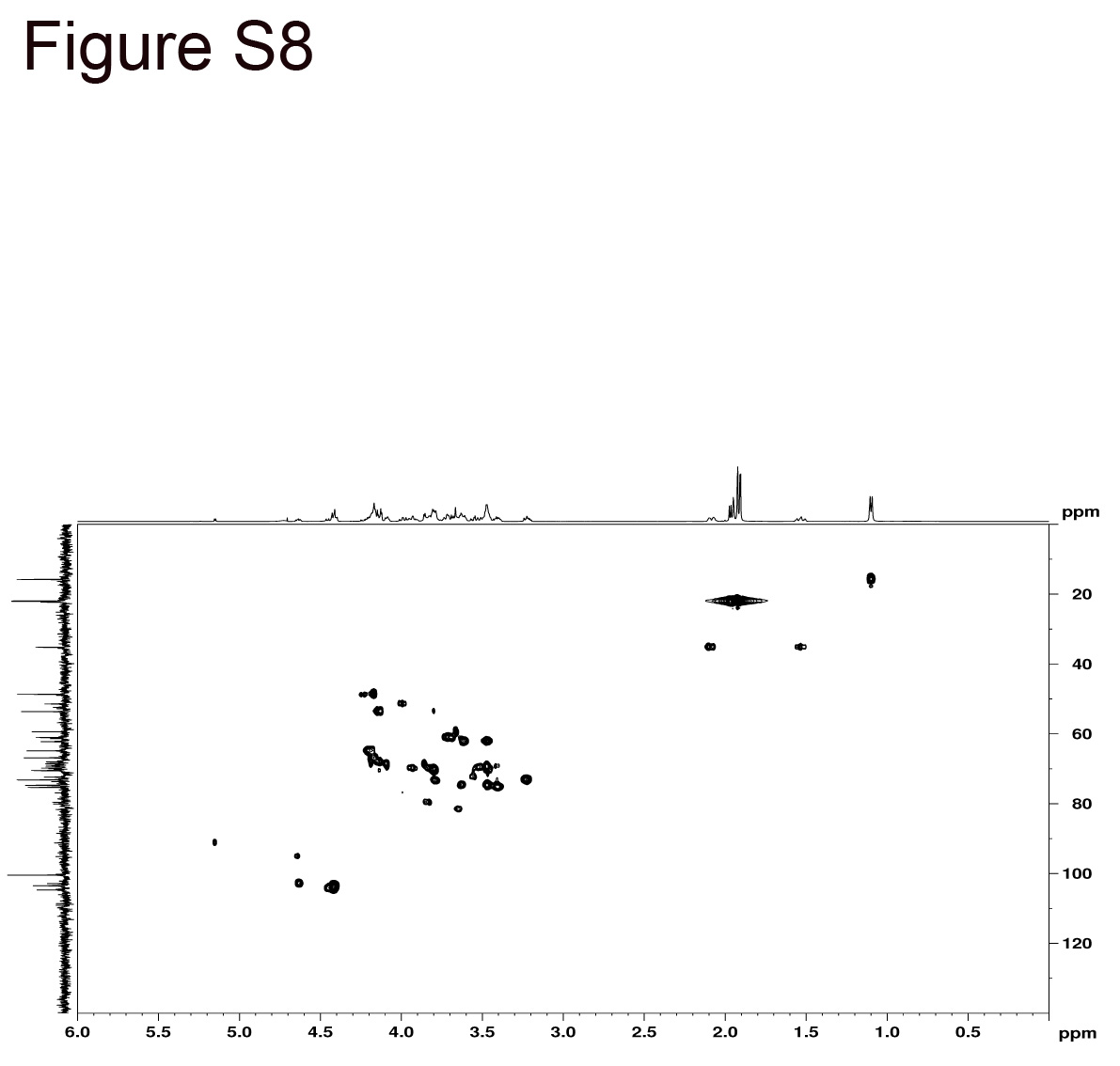
**Supplementary Figure 7. HSQC spectrum (500 MH_Z_, D_2_O) of ΦAB6 TSP-digested products of *Ab*-54149 exopolysaccharide.**


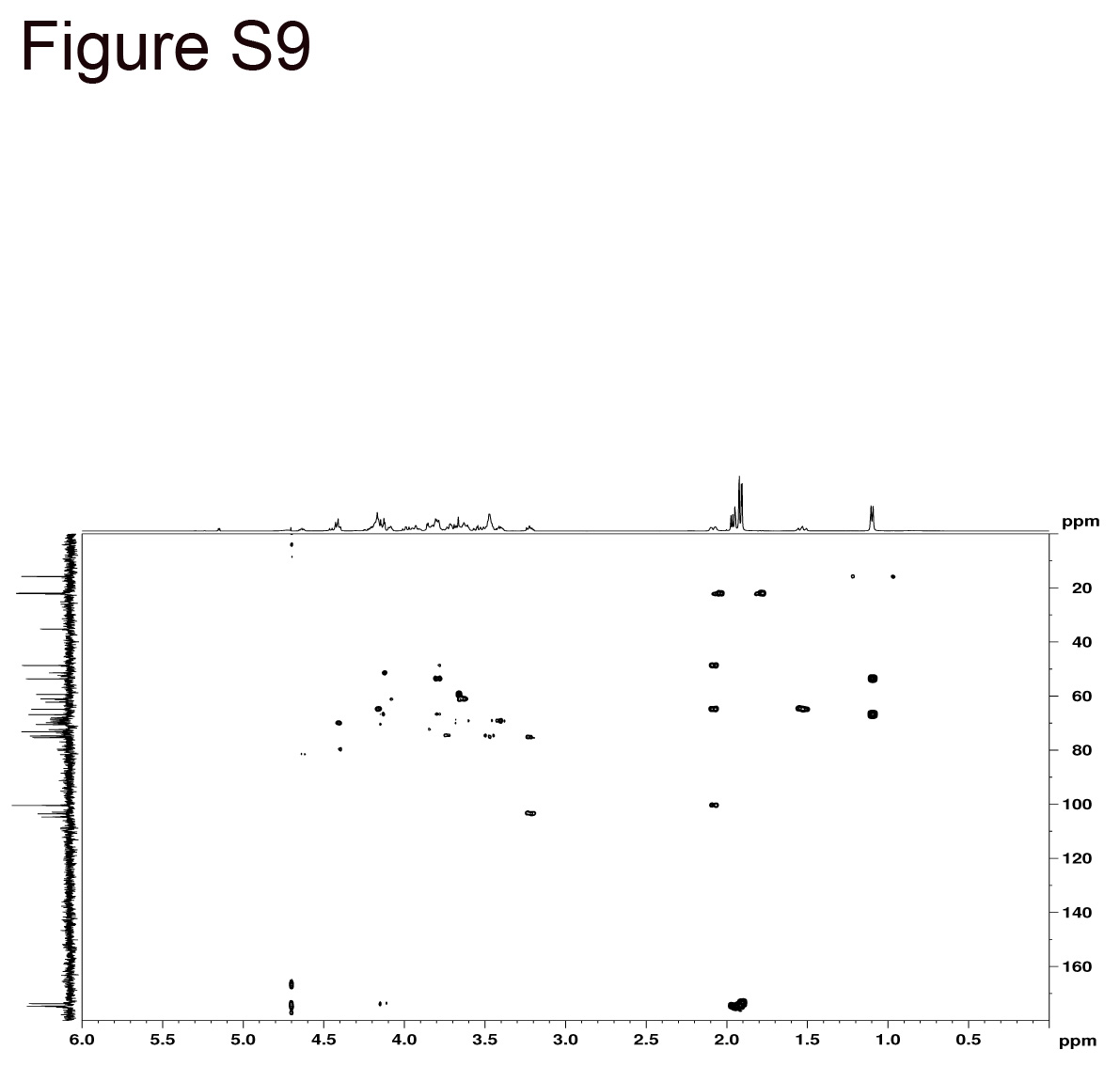
**Supplementary Figure 8. HMBC spectrum (500 MH_Z_, D_2_O) of ΦAB6 TSP-digested products of *Ab*-54149 exopolysaccharide.**


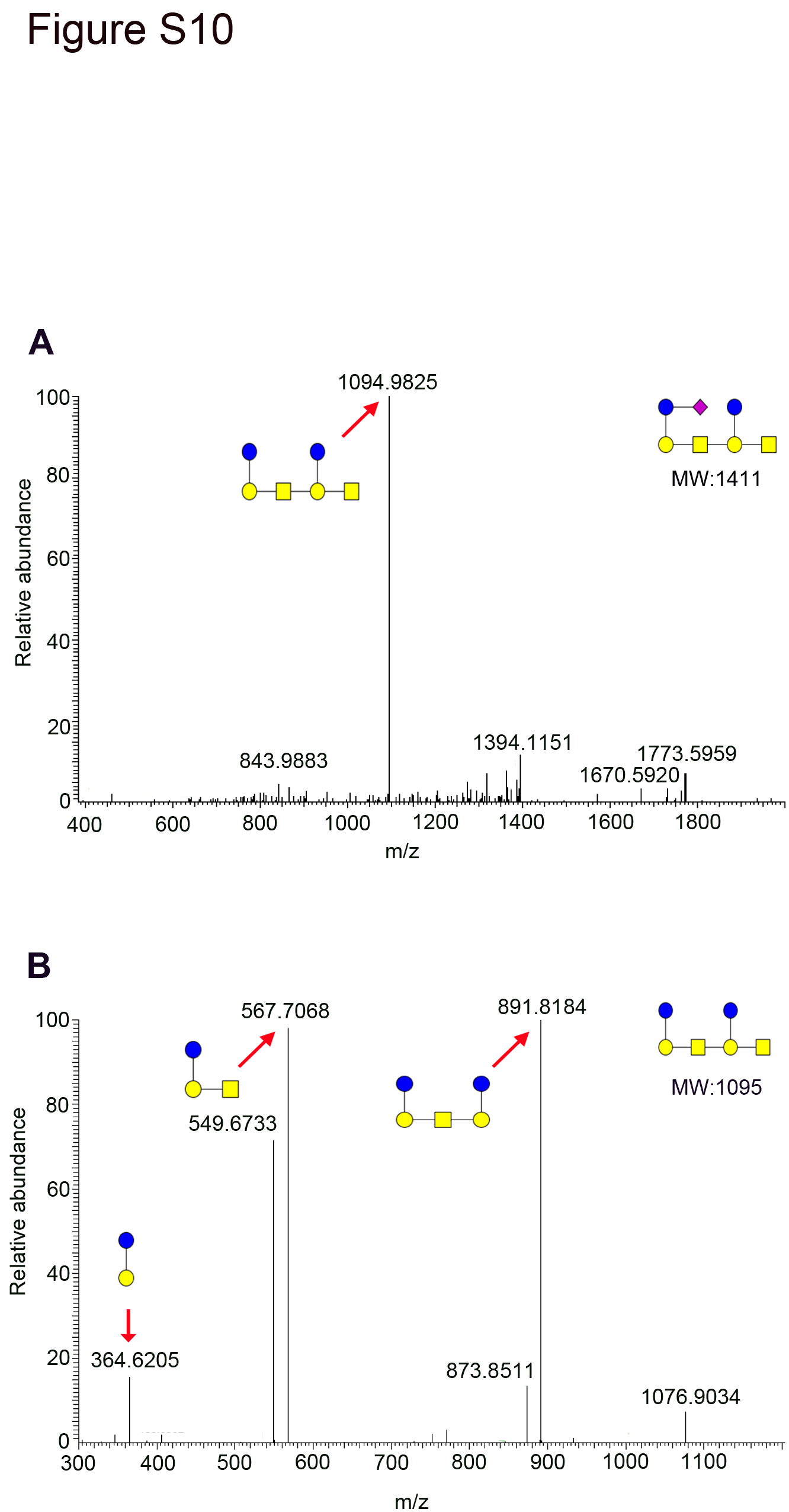


**Supplementary Figure 9. LC-ESI-MS-MS analysis of the two minor ΦAB6 TSP-digested products of *Ab*-54149 exopolysaccharide.** The two minor peaks from the LC-ESI-MS spectrum (Fig. 2), corresponding to the m/z values 1411.50 and 1095.51, respectively, were analyzed. The representation of sugars is the same as in Fig. 2.

**
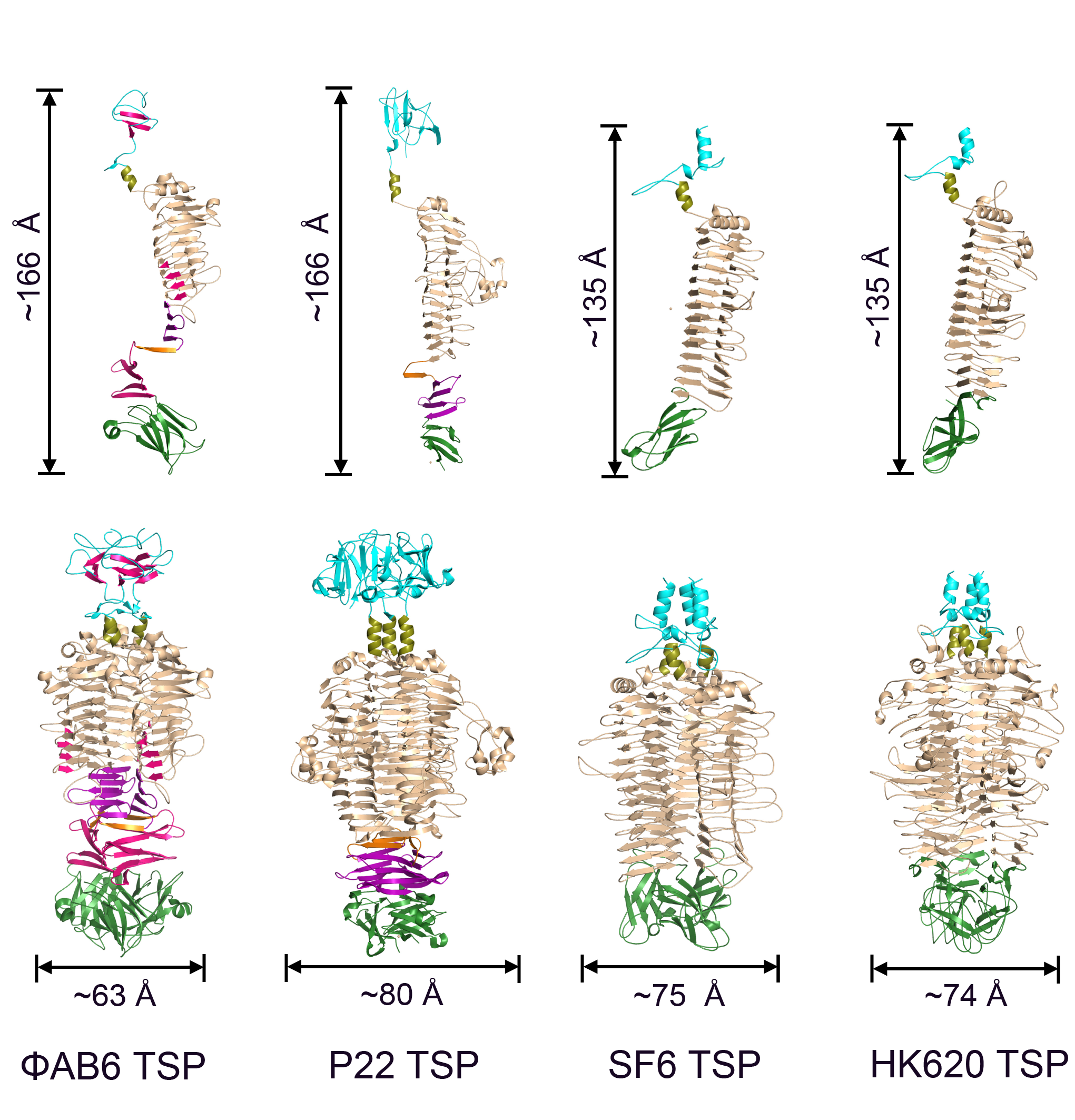
**

**Supplementary Figure 10. Structural comparison of ΦAB6 TSPΔN, P22 TSP, SF6 TSPΔN, and HK620 TSPΔN.** The N-terminal domain, linker region, β-helix domain, and the C-terminal domain in these structures are colored cyan, deep olive, wheat, and dark green, respectively. The triangular β-prisms and the interdigitated segment in ΦAB6 TSPΔN and P22 TSP are colored purple, hot pink, or orange. The PBD β-sheet and the β-helix B1a strands in ΦAB6 TSPΔN are also highlighted in hot pink.

**
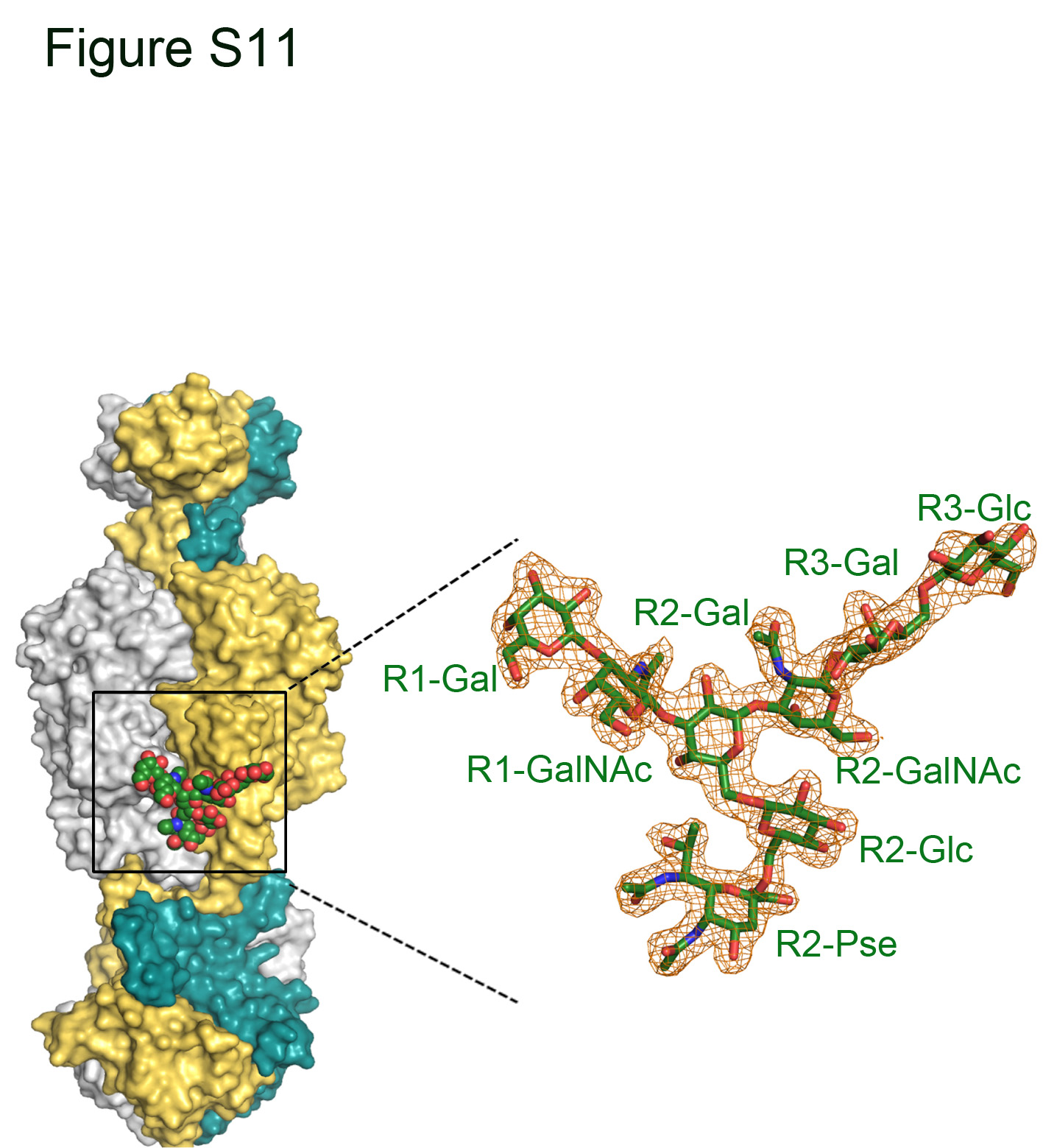
**

**Supplementary Figure 11.** Crystal structure of ΦAB6 TSP∆N in complex with the oligosaccharides of three-repeated units. This complexed structure was ontained by soaking the ΦAB6 TSP∆N crystals in reservoir solution containing the crude extract of *Ab*-54149 exopolysaccharide for 15 hours. The three subunits of the enzyme are colored white, olive, and deepteal, respectively. The oligosaccharides bound to the intersubunit carbohydrate-binding grooves of the enzyme are shown with sphere model. The figure on the right-hand side shows the 1σ 2*F_o_-F_c_* electron density map around the bound oligisaccharides overlaid with the final refined model.

**
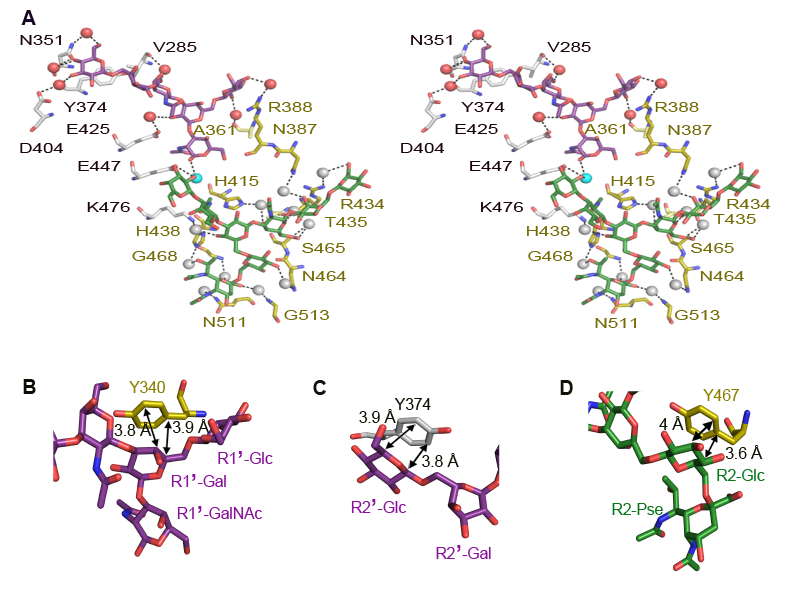
**

**Supplementary Figure 12.** Interactions between ΦAB6 TSP and the bound oligosaccharides. (A) The water-mediated hydrogen bonds between the enzyme and the bound oligosaccharide. The orientation and color codes of the figure are the same as those in Fig. 5C. The water molecules involved in binding to the R1’-R2’ and R1-R2-R3 fragment are colored red and gray, respectively. The water molecule involved in both fragments is colored cyan. (B) The CH/π interaction between Tyr340 and R1’-Gal*p*. **(**C**)** The CH/π interaction between Tyr374 and R2’-Glc*p*. **(**D**)** The CH/π interaction between Tyr467 and R2-Glc*p*

**
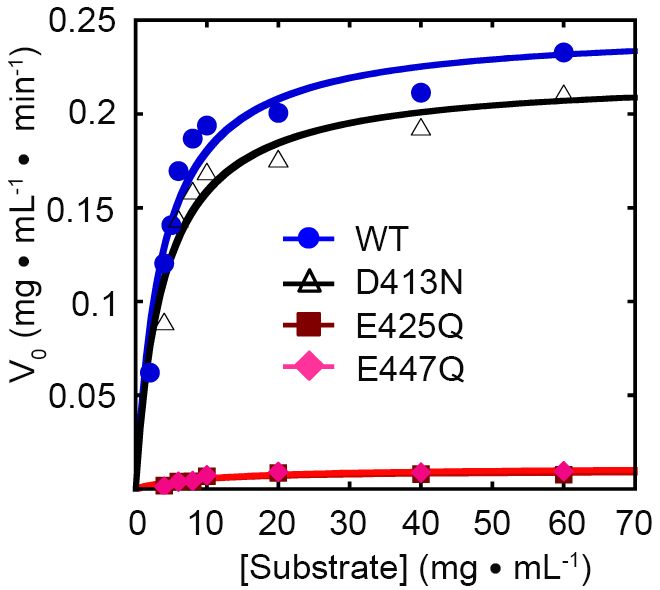
**

**Supplementary Figure 13. Enzyme kinetic assay of wild-type and mutant ΦAB6 TSP.**

**
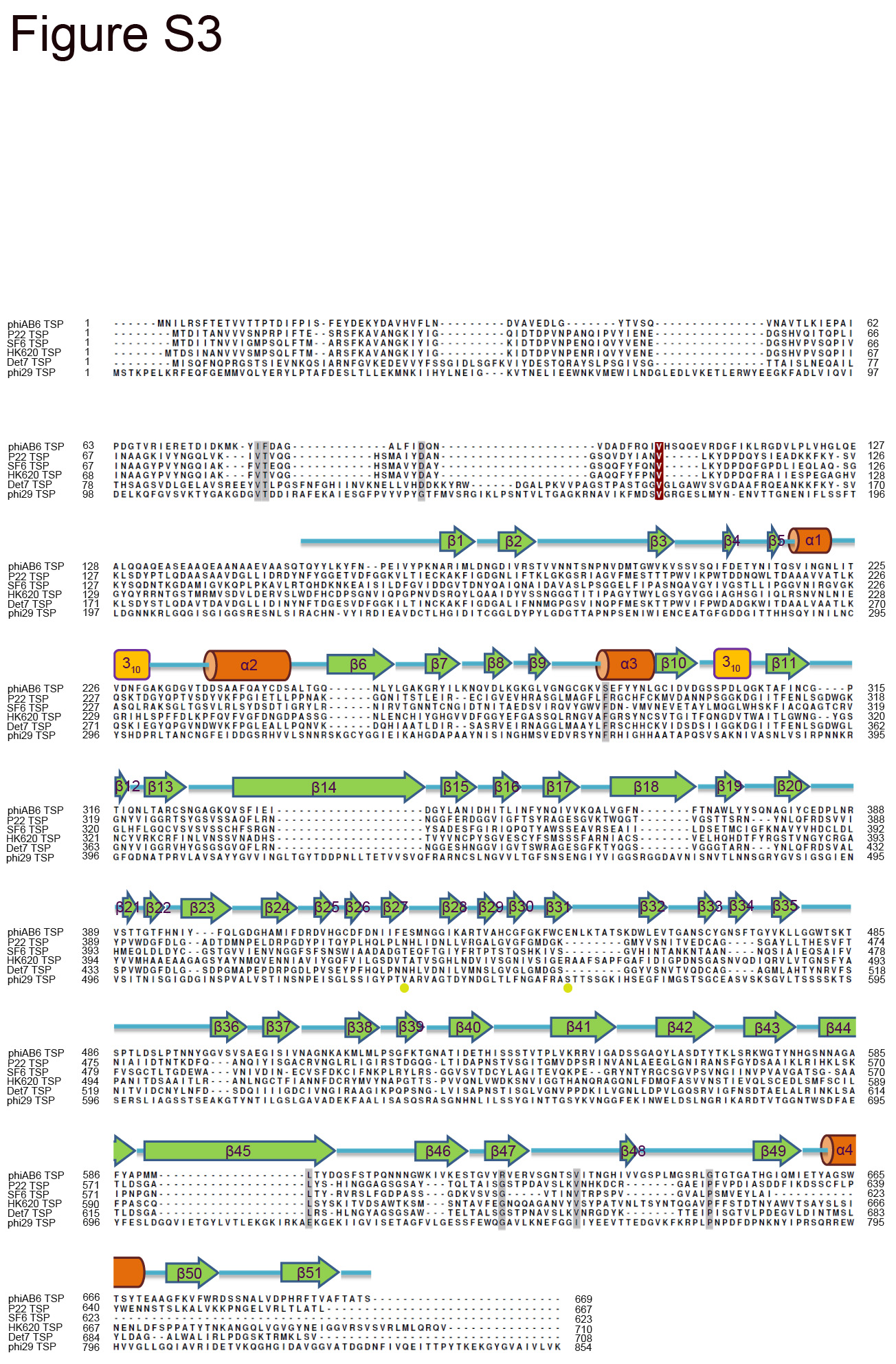
**

**Supplementary Figure 14. Structure-based sequence alignment of ΦAB6 TSP with other representative bacteriophage TSPs.** The secondary structural elements are illustrated according to the refined structure of ΦAB6 TSP**ΔN**. The residues that are conserved in five of the six sequences are shaded in gray. The completely conserved residues are highlighted in brown. The yellow balls depict the proposed catalytic residues of ΦAB6 TSP.

**
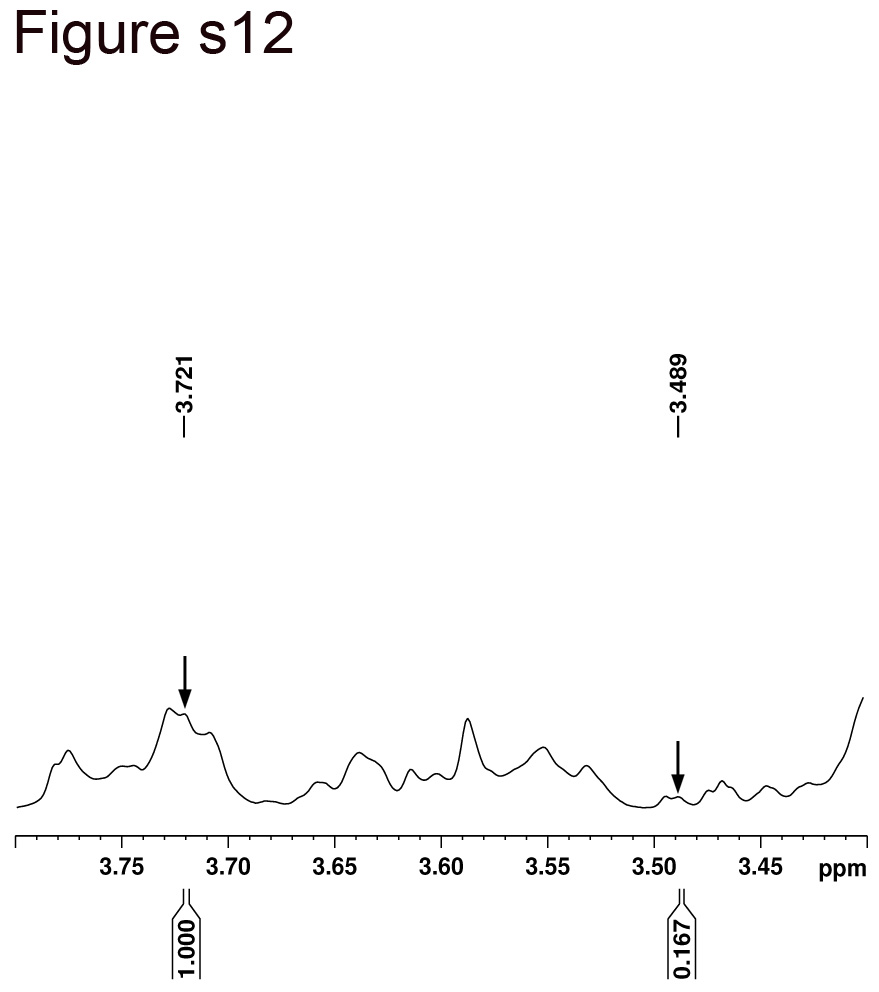
**

**Supplementary Figure 15. Quantification of pseudaminic acid content of *Ab*-54149 exopolysaccharide.** The quantification was based on a NMR spectrum of whole exopolysaccharide extract. The black arrows indicate the chemical shifts **o**f H-6 at the branched glucose with (3.72 ppm) or without (3.48 ppm) the bound pseudaminic acid, respectively. The integral values for the areas of the two peaks are shown underneath the spectrum.

**Supplementary Table**

**Table 1.** The proton (^1^H) and carbon (^13^C) chemical shifts on NMR spectra of ΦAB6 TSP-digested products of *Ab*-54149 surface polysaccharide.

|  |  | 1 | 2 | 3 | 4 | 5 | 6 |  |
| --- | --- | --- | --- | --- | --- | --- | --- | --- |
| A | ^1^H | 4.63 | 3.98 | 3.83 | 4.09 | 3.63 | 3.71 |  |
| 3-β-GalNAc | ^13^C | 102.9 | 51.4 | 79.6 | 68 | 74.8 | 61.1 |  |
| B | ^1^H | 4.46 | 3.52 | 3.66 | 4.1 | 3.81 | 3.83; 3.93 |  |
| 3,6-β-Gal | ^13^C | 104.7 | 69.3 | 81.7 | 68.5 | 73.7 | 70.8 |  |
| C | ^1^H | 4.41 | 3.23 | 3.42 | 3.5 | 3.64 | 3.72;3.6 |  |
| 6-β-Glc | ^13^C | 104 | 73.2 | 75.3 | 70.2 | 74.6 | 61.1 |  |
| C’ | ^1^H | 4.39 | 3.21 | 3.41 | 3.38 | 3.46 | 3.48; 3.62 |  |
| t-β-Glc | ^13^C | 104.2 | 73.3 | 75.3 | 69.4 | 73.9 | 61.2 |  |
|  |  | 3 | 4 | 5 | 6 | 7 | 8 | 9 |
| D | ^1^H | 2.08; 1.53 | 4.19 | 4.17 | 3.8 | 4.13 | 4.15 | 1.1 |
| t-α-Pse | ^13^C | 35.4 | 64.9 | 48.7 | 70 | 53.5 | 67 | 15.7 |
